# Cerebral white matter hyperintensity volumes: Normative age- and sex-specific values from 15 population-based cohorts comprising 14,876 individuals

**DOI:** 10.1016/j.neurobiolaging.2024.11.006

**Published:** 2024-11-20

**Authors:** Floor A.S. de Kort, Elisabeth J. Vinke, Ewoud J. van der Lelij, Devasuda Anblagan, Mark E. Bastin, Alexa Beiser, Henry Brodaty, Nishi Chaturvedi, Bastian Cheng, Simon R. Cox, Charles DeCarli, Christian Enzinger, Evan Fletcher, Richard Frayne, Marius de Groot, Felicia Huang, M. Arfan Ikram, Jiyang Jiang, Bonnie Y.K. Lam, Pauline Maillard, Carola Mayer, Cheryl R. McCreary, Vincent Mok, Susana Muñoz Maniega, Marvin Petersen, Genady Roshchupkin, Perminder S. Sachdev, Reinhold Schmidt, Stephan Seiler, Sudha Seshadri, Carole H. Sudre, Götz Thomalla, Raphael Twerenbold, Maria Valdés Hernández, Meike W. Vernooij, Joanna M. Wardlaw, Wei Wen, Hugo J. Kuijf, Geert Jan Biessels, J. Matthijs Biesbroek

**Affiliations:** a Department of Neurology, University Medical Center Utrecht Brain Center, Utrecht, the Netherlands; b Department of Radiology & Nuclear Medicine, Erasmus MC University Medical Center, Rotterdam, the Netherlands; c Department of Epidemiology, Erasmus MC University Medical Center, Rotterdam, the Netherlands; d Centre for Clinical Brain Sciences, University of Edinburgh, Edinburgh, UK; e Department of Biostatistics, Boston University, Boston, USA; f Centre for Healthy Brain Ageing (CHeBA), Discipline of Psychiatry and Mental Health, School of Clinical Medicine, UNSW Sydney, Sydney, Australia; g MRC Unit for Lifelong Health and Ageing, University College London, London, UK; h Department of Neurology, University Medical Hospital Hamburg-Eppendorf, Hamburg, Germany; i Lothian Birth Cohorts, Department of Psychology, University of Edinburgh, Edinburgh, UK; j Department of Neurology, University of California Davis, Sacramento, USA; k Department of Neurology, Medical University Graz, Graz, Austria; l Department of Clinical Neurosciences, University of Calgary, Calgary, Canada; m Department of Radiology, University of Calgary, Calgary, Canada; n Division of Neurology, Department of Medicine and Therapeutics, Faculty of Medicine, The Chinese University of Hong Kong, China; o Glenn Biggs Institute for Alzheimer’s & Neurodegenerative Diseases, University of Texas Health Science Center, San Antonio, USA; p Centre for Medical Image Computing, University College London, London, UK; q School of Biomedical Engineering & Imaging Sciences, King’s College London, UK; r Department of Cardiology, University Heart & Vascular Center Hamburg, University Medical Center Hamburg-Eppendorf, Hamburg, Germany; s DZHK (German Centre for Cardiovascular Research) partner site, Hamburg, Germany; t UK Dementia Research Institute, University of Edinburgh, Edinburgh, UK; u Image Sciences Institute, University Medical Center Utrecht, Utrecht, the Netherlands; v Department of Neurology, Diakonessenhuis Hospital, Utrecht, the Netherlands

**Keywords:** Cerebral small vessel disease, White matter hyperintensities, Normative data, Population-based, MRI, Aging

## Abstract

White matter hyperintensities (WMH) increase with age, with marked interindividual variation. There is a need for normative data by age and sex, to improve individualized WMH burden assessment. In this study, we pooled cross-sectional data from 15 population-based cohorts (14,876 nondemented individuals, age 18–97 years), through the Meta VCI Map consortium. Whole brain and tract-specific MRI-assessed WMH volumes were calculated in MNI-152 space. We used quantile regression to create centile curves of WMH volume versus age, stratified by sex. Total WMH volume and interindividual variance increased exponentially with age for both sexes, with females showing higher WMH volumes. WMH volume increase with aging was not uniform across the white matter, but instead followed one of three different patterns depending on location. Age- and sex-specific normative data for total and regional WMH volumes were created. Our study provides detailed information on the normal distribution of total and regional WMH volumes across adulthood. The normative data enable a quantitative approach to interpreting total and regional WMH volumes in clinical practice and research settings.

## 1. Introduction

Cerebral small vessel diseases (SVDs) are a major cause of morbidity with aging (Markus and de Leeuw, 2023). Approximately one in four ischemic strokes and most hemorrhagic strokes in older people are due to SVDs (Wardlaw et al., 2019). Moreover, SVDs contribute to the development of dementia in a substantial proportion of individuals (Wardlaw et al., 2022). The most common manifestation of SVD on brain MRI are white matter hyperintensities (WMH) of presumed vascular origin (Duering et al., 2023). The prevalence and burden of WMH increase with age. In fact, over the age of 70 it is uncommon to have a brain MRI scan without any WMH (Smith et al., 2017b). Many individuals with WMH, even at substantial volumes, can be without evident symptoms (Wardlaw et al., 2021). Yet, both in asymptomatic and in symptomatic individuals, the burden of WMH is predictive of adverse outcomes. A systematic review reported elevated risks of dementia, incident stroke and death in people with extensive WMH burden (Debette et al., 2019).

Despite WMH being highly prevalent with aging and an important predictor of poor health outcomes, literature on normal distribution of WMH across adulthood is scarce and based on single-center studies, limiting the generalizability of findings (Fatemi et al., 2018; Garnier-Crussard et al., 2020; Habes et al., 2018b, 2016; Huang et al., 2018; King et al., 2014; Lohner et al., 2022; Yang et al., 2016). Additionally, precise estimates of age-specific normal variance in WMH volumes are lacking. Consequently, it can be challenging to determine whether an individual’s burden of WMH falls within or outside the ‘normal’ range for age, also because of marked interindividual variability (Debette et al., 2019; Smith et al., 2017b). This can generate substantial diagnostic uncertainty. Hence, there is a need for widely applicable age-specific normative data from the general population for WMH volumes, also taking into account male to female differences (Lohner et al., 2022). This may aid in a better interpretation of MRI results, informing patients, and identifying individuals with extensive WMH burden who might benefit from proposed strategies to prevent adverse clinical outcomes (Smith et al., 2017a; Wardlaw et al., 2022). It can also be of value for SVD research. Normative data should also consider regional distributions of WMH, as there is evidence that location of WMH in specific white matter regions may impact cognitive performance and might well be relevant for other outcomes (Coenen et al., 2023). Moreover, regional distributions may be indicative of different underlying etiologies (Biesbroek et al., 2024).

Through the Meta VCI Map consortium, we have harmonized MRI-data from 15 population-based studies, comprising a total of 14,876 individuals, to perform lesion symptom mapping studies (Weaver et al., 2019). These harmonized data cover a wide age range, with equal representation of both sexes, and are geographically diverse. Here, we used this resource to deliver estimates of normative WMH volumes, also considering interindividual heterogeneity and created age- and sex-specific reference data for total and regional WMH volumes. Such data can support an individualized quantitative WMH assessment.

## 2. Material and Methods

### 2.1. Subject selection

We harmonized and pooled individual person data from 15 cohorts across eight countries spanning four continents: Australia (Sydney Memory and Ageing Study (MAS) and the Older Australian Twins Study (OATS)), Austria (Austrian Stroke Prevention Study (ASPS) and the Austrian Stroke Prevention Family Study (ASPSF)), Canada (Calgary Normative Study (CNS)), China (Chinese University of Hong Kong- Risk Index for Subclinical brain lesions in Hong Kong (CU-RISK)), Germany (Hamburg City Health Study (HCHS)), the Netherlands (Rotterdam Study (RS)), the UK (Southall And Brent Revisited (SABRE) and the Lothian Birth Cohorts 1921 (LBC1921) and 1936 (LBC1936)), and the USA (Framingham Heart Study Offspring cohort (FHS_Gen2); Third generation cohort (FHS_Gen3); Minorities cohort (FHS_Omni1) and the UC Davis Alzheimer’s Disease Center Diversity Cohort (AUCD)) through the Meta VCI Map consortium. Cohort-specific details are provided in the supplemental material (Table S1). Background and organization of the Meta VCI Map consortium are described in a design article (Weaver et al., 2019) and on the consortium website www.metavcimap.org. Cohorts were eligible for inclusion if participants were recruited from the general population and underwent brain MRI (with availability of FLAIR and T1 sequences). Each participant contributed only one scan timepoint. This timepoint was selected on a per-cohort basis, details in Table S1. Individuals with dementia were excluded, but not those with mild cognitive impairment or a history of stroke. Next, individuals with missing data on dementia diagnosis, age or sex were excluded. The flowchart of the final participant selection is shown in Figure S1. For all cohorts, ethical and institutional approval was obtained as required by local regulations to allow data acquisition, including informed consent, and data sharing.

### 2.2. Harmonization of subject characteristics

Age was rounded to whole years. Sex reflects ‘sex assigned at birth’ for all cohorts, henceforth referred to as sex. All harmonization steps are described in detail in the supplemental material. Missing data on baseline characteristics primarily result from differences in the acquisition of these parameters across cohorts (e.g., only a subset of cohorts collected data on all cardiovascular risk factors). A cohort-specific baseline table is provided in Table S2 and specific reasons for missing data by cohort are detailed in the design papers referenced in Table S1.

### 2.3. Image processing

Cohort-specific details including MRI acquisition, image processing protocols, and quality control are described in the supplemental material. In short, WMH segmentations were provided by the participating centers for 14 cohorts. The participating centers ensured quality control of these segmentations. For one cohort, WMH maps were automatically computed at the UMC Utrecht, The Netherlands (Kuijf et al., 2019). WMH maps were registered to the 1×1×1 mm Montreal Neurological institute (MNI)-152 brain template for spatial normalization (Fonov et al., 2011). Ten cohorts shared WMH maps that were already in MNI-152 space. World coordinates and/or voxel sizes were adjusted to match the 1×1×1 mm resolution MNI-152 brain template where appropriate. For the remaining five cohorts, registration was performed centrally using RegLSM (Biesbroek et al., 2019). The normalization procedure corrects for differences in head size and brain volume. To minimize effects of possible misclassifications of other lesion types such as cortical or large subcortical infarcts as WMH during the WMH segmentation, voxels located outside the white matter (defined as a probability below 30 % according to the MNI probabilistic white matter atlas) were removed from all individual WMH maps. Final WMH prevalence maps for the pooled cohort, stratified by sex, are shown in Figure S2. The fully processed WMH maps were used to calculate normalized total (whole brain) WMH volumes and regional WMH volumes in all 20 white matter (WM)-tracts included in the JHU atlas (thresholded at 10 %) (Hua et al., 2008). All normalized WMH volumes (henceforth referred to as WMH volumes) were log10-transformed prior to further analysis. Since volumes of 0 mL cannot be log-transformed, 0.001 mL (equivalent to one voxel in MNI-152 space) was added to each volume beforehand. Figure S3 shows examples of WMH maps corresponding to specific log10-transformed volumes, as a visual assistance to interpret WMH volumes. As a reference, the intracranial volume of the MNI-152 template is 1669.67 mL.

### 2.4. Data analysis

All analyses were based on cross-sectional data and were stratified for sex.

#### 2.4.1. Exploratory analyses

Due to the heterogeneity in imaging acquisition and processing, as well as in inclusion and exclusion criteria among the cohorts, we first explored potential differences and compared the distributions of log10-transformed WMH volume in relation to age across cohorts, before merging all data to perform normative modeling. Initially, Plotly Express in Python (https://plot.ly) was used to create scatterplots stratified by cohort, facilitating the visual inspection of the relationship between log10-transformed WMH volume and age, as well as to compare distributions among cohorts. Additionally, we stratified data by 5-year age intervals to analyze data distribution. For the two most represented 5-year age strata, we compared median WMH volumes and interindividual variability between the total sample and the two largest cohorts (details in supplemental material).

#### 2.4.2. Main analyses

Quantile regression was used for normative modeling of WMH volume versus age. This approach was chosen because of the robustness of quantile regression to outliers, and the possibility to take into account non-linear age terms (Koenker and Bassett, 1978; Onyedikachi, 2015). The smoothness of the fitted curves is influenced by the degrees of freedom (*δ)*, a user-defined parameter. To account for possible nonlinear percentiles, exploratory analyses were performed to assess whether splines of age improved the model compared with the linear age term. To select the optimal parameters for the age splines, we used ANOVA to assess whether the model improved with increasing degrees of freedom, followed by a visual assessment of the curves to prevent overfitting (i.e., to avoid capturing noise). As a result, splines of age with four degrees of freedom were used for total WMH volume and with two degrees of freedom for tract-specific WMH volumes. The combination of using age splines and the disjointed age ranges between cohorts precluded correction for study site in the quantile regression models. To assess the precision of the fitted curves (10th, 50th, and 90th centile curves), we performed a bootstrapping procedure with 1000 iterations, randomly sampling participants with replacement and re-estimating the centile curves. A distribution of possible curves was collected from which 95 % confidence intervals were estimated. All analyses were performed using the packages quantreg, splines and boot in R (v4.1.2) (https://cran.r-project.org).

To assess whether differentiating patterns for the 20 white matter tracts could be identified, all tract-specific plots were qualitatively assessed by visual inspection of the p10, p50, and p90 curves, considering the age at which WMH accumulation started and the shape and steepness or the normative curves.

#### 2.4.3. Sensitivity analyses

We conducted two sensitivity analyses. In the first analysis, we replicated the quantile regression-based normative modeling excluding individuals with a history of stroke, as these individuals may have larger WMH volumes, potentially affecting the results. In the second sensitivity analysis, the quantile regression models were adjusted for image registration method, with RegLSM, employed in 7 out of 15 cohorts, as the reference. This analysis aimed to assess the effect of heterogeneity in image processing pipelines on the results.

## 3. Results

### 3.1. Sample characteristics

We analyzed 14,876 individuals from 15 population-based cohorts from North America, Europe, Asia and Oceania. Three cohorts targeted ethnic diversity in their recruitment (Table S1). Yet, 88.3 % (n=13,014) of the overall cohort was of white race and ethnicity. There was a near equal representation of both sexes (overall 52.2 % (n=7762) female). The targeted age range varied substantially across cohorts (Table S1). In the pooled cohort, each sex-specific 5-year age stratum was represented by at least 160 participants for the ages of 40–85 years, with more than 1400 participants included for ages 70–75 years. A detailed overview of the number of participants for each sex-specific 5-year age-stratum is provided in the supplemental information. Overall, mean age was 63.9 years (SD 11.9) and comparable between both sexes. Table 1 shows baseline characteristics stratified by sex and Table S2 stratified by cohort.

### 3.2. Distribution of WMH volumes in the individual cohorts and the pooled cohort

**Fig. 1.**
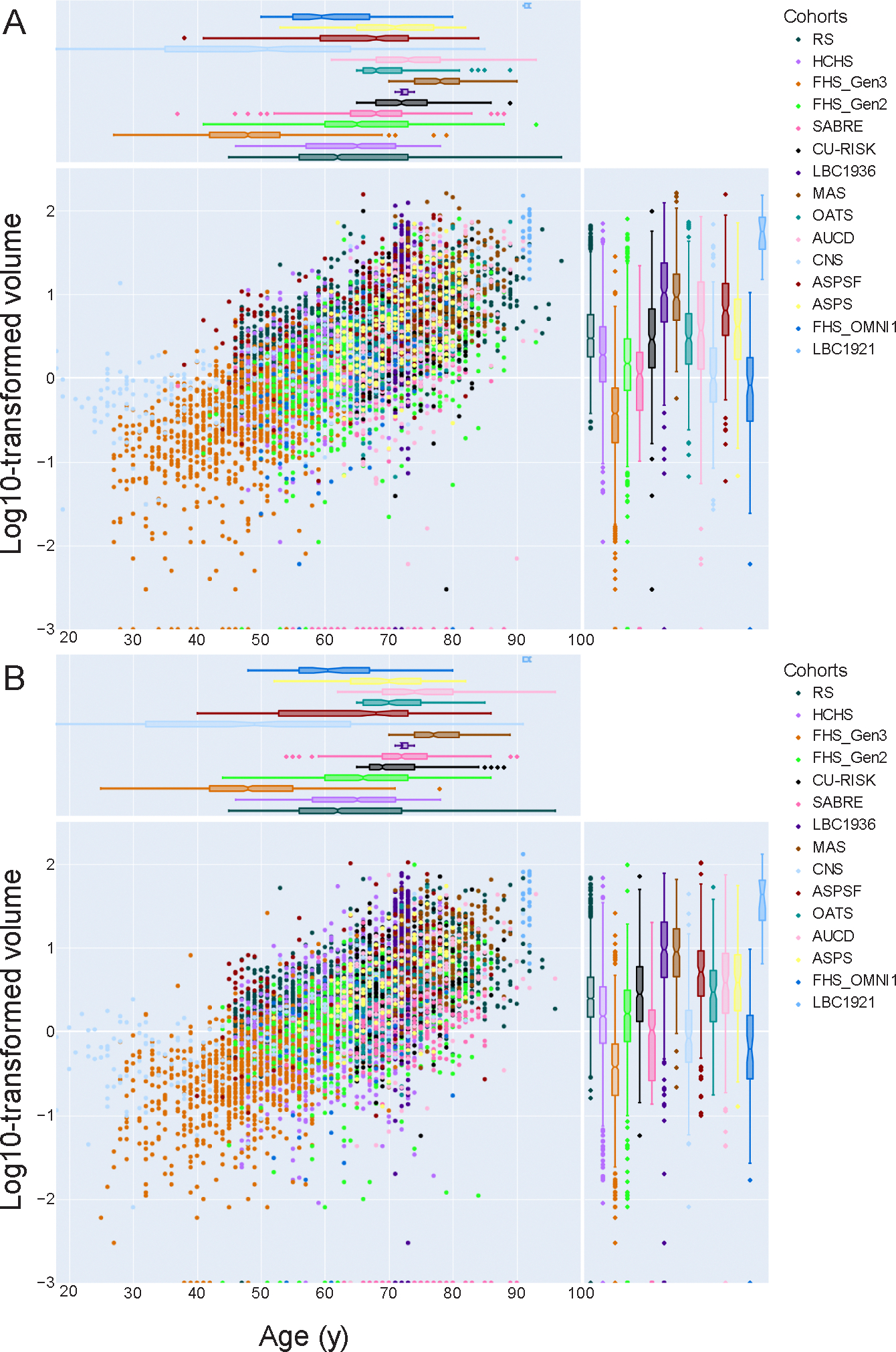
Cohort-specific distribution of total white matter hyperintensity volumes in relation to age for females (A) and males (B). Main panels: scatterplot of log10-transformed white matter hyperintensity volume in milliliter versus age, stratified by cohort. Side panels: cohort specific distribution of age (upper panel) and white matter hyperintensity volume (right-sided panel). Datapoints of all cohorts were plotted on top of each other, based on sample size of cohort (topdown). Color-mapping was based on the 15-color palette for color blindness by Martin Krzywinski, accessed via https://mk.bcgsc.ca/biovis2012/ on June 20th, 2023.

Figure 1 shows that scatterplots of log10-transformed WMH volumes versus age largely overlapped between the 15 cohorts and Figure S4 shows scatterplots for each cohort, illustrating the overlap between datapoints from individual cohorts and the median WMH volumes of the pooled cohort. In the two largest cohorts (RS, HCHS), median WMH volumes varied, on average, by 10–25 % from the pooled cohort median for the 65–69 and 70–74 age groups. Moreover, the interindividual variation within each cohort showed slight differences but was generally consistent with the variation observed in the pooled cohort. For instance, in females aged 60–64 years, the 75th percentile was 2.5-fold (RS), 4.1-fold (HCHS), and 3.7-fold (pooled cohort) the WMH volumes at the 25th percentile (see supplemental information for details). In addition, Figure S4 also shows that patterns were largely comparable between cohorts comprising individuals of 100 % non-white races and ethnicities, such as CU-RISK, and cohorts with (predominantly) white populations.

### 3.3. Normative centile curves

#### 3.3.1. Normative centile curves for total WMH volumes

**Fig. 2.**
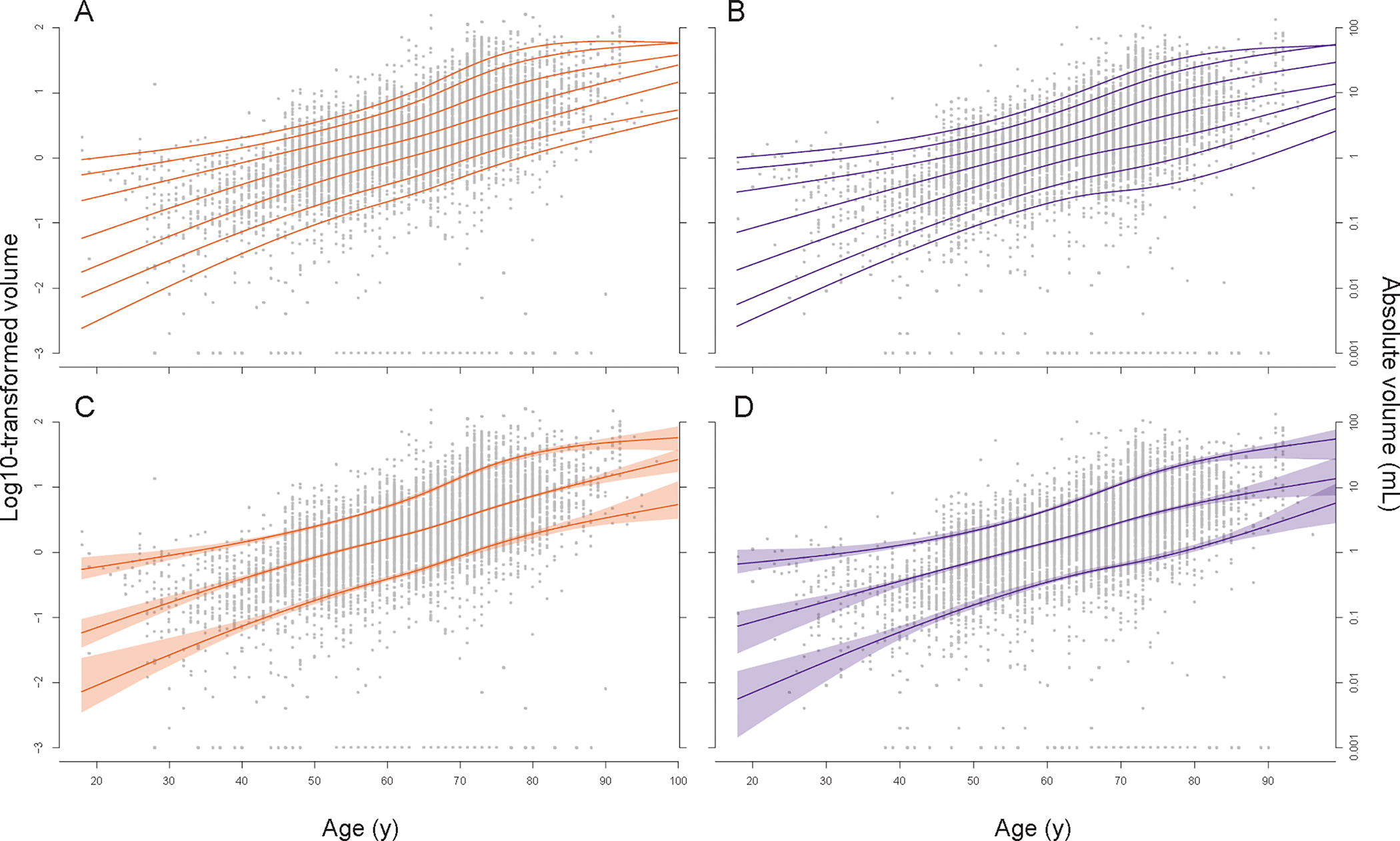
Centile curves for total log10-transformed white matter hyperintensity volume, stratified by sex. Upper panels: 5th, 10th, 25th, 50th, 75th, 90th, and 95th centile curves for females (A) and males (B) respectively. Lower panels: corresponding 95 % confidence intervals for the 10th, 50th and 90th centile curves for females (C) and males (D) respectively. Abbreviations: mL, milliliter; y, years. Color-mapping was based on contrasting colors for color blindness by David Nichols, accessed via https://davidmathlogic.com/colorblind on June 20th, 2023.

The derived centile curves for the pooled cohort showed narrow 95 % confidence intervals for the 10th, 50th and 90th centiles for the ages 40–85 years. The median (50th percentile) curve for log10-transformed WMH volume showed an approximately linear increase across ages (Figure 2). Accordingly, the corresponding absolute volumes showed an exponential increase, as shown in Table 2 and Figure S5, with doubling in approximately every 10 years of age in both sexes. On average, females showed higher median WMH volumes than males starting from the age of 40 onward. This difference was equivalent to the effect of two years of aging between the ages of 40 and 70, and up to five years at older ages (above 80 years).

In both sexes, there was marked interindividual variance in WMH volumes at each age and this variance increased exponentially with aging (illustrated at the curves of non-log10-transformed volumes, Figure S5). In addition, Figure 2 shows that the distances between the 10th, 25th, 50th, 75th and 90th centile curves of the log10-transformed volumes were relatively stable across ages (with a slight increase for the ages 65–80 years). The absolute volumes at the 75th percentile were approximately four times (3.5 to five times) the volumes at the 25th percentiles across ages and sexes. Thus, the 25th percentile at a given age was like the 50th percentile of individuals being 10 years younger, and the 75th percentile to that of individuals aged approximately 10 years older.

#### 3.3.2. Sensitivity analyses

The sensitivity analyses excluding those with prior stroke showed that the median WMH volumes were, on average, 1–2 % lower in comparison to the unselected pooled cohort (Figure S6, Table S3). The sensitivity analyses adjusted for image registration method, showed similar patterns to the original models, with a linear increase of the log10-transformed median curves and comparable interindividual variation, although the 5th centile curves were slightly less stable. Median WMH volumes differed by about 15 % from the original model (Figure S7, Table S4). Further details are in the supplemental material.

#### 3.3.3. Normative centile curves for regional WMH volumes

**Fig. 3.**
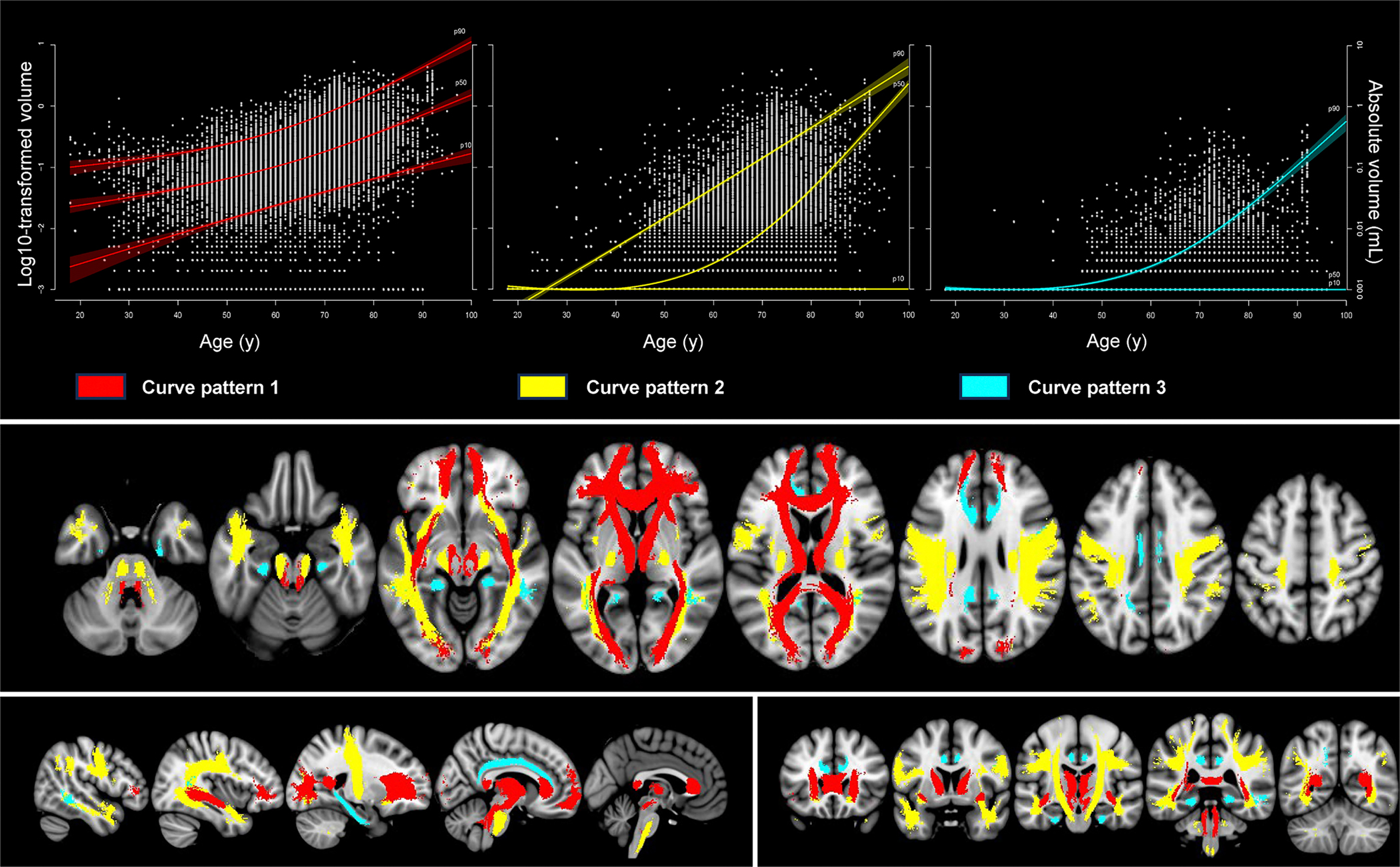
Three identified percentile curve patterns for tract-specific white matter hyperintensity volumes. Upper panel: this figure shows examples of the three identified curve patterns of tract-specific white matter hyperintensity volumes with aging. From left to right, respectively the anterior thalamic tract right (in red), as an example of curve pattern 1 (tracts following the same pattern as total white matter hyperintensity volume), the inferior longitudinal fasciculus left (in yellow) as an example of curve pattern 2 (tracts where white matter hyperintensity accumulation started at the age of 60–65 years and accelerated at higher ages) and the cingulum cingulate gyrus right (in blue) as an example of curve pattern 3 (tracts where white matter hyperintensities were rare even at higher ages). The curves reflect the 10th, 50th and 90th centile curves and corresponding 95 % confidence intervals. Note that for curve pattern 3, both the 10th and 50th centile curves never left baseline. Lower panel: all 20 white matter tracts projected onto the MNI-152 template, color-coded according to their curve pattern. Tracts following curve pattern 1 in red (forceps major, forceps minor, anterior thalamic radiation and inferior fronto-occipital fasciculus), tracts following curve pattern 2 in yellow (superior longitudinal fasciculus, inferior longitudinal fasciculus, corticospinal tract and uncinate fasciculus), and tracts following curve pattern 3 in blue (superior longitudinal fasciculus temporal part, cingulum cingulate gyrus, cingulum hippocampus). Projections are in transversal (upper figure), sagittal (lower figure-left) and coronal (lower figure-right) plain.

Normative curves for regional WMH volumes in all 20 WM-tracts with aging were largely comparable between right- and left-sided tracts. We identified three different curve patterns for the tracts over time. Figure 3 illustrates the location of these tracts on the MNI-152 template and provides an example for each pattern. Figure S8 provides a complete overview of all tracts, stratified by sex. The first curve pattern followed total WMH volume with aging. This pattern was seen for the forceps major (FMaj), forceps minor, anterior thalamic radiation (ATR) and inferior fronto-occipital fasciculus; tracts mostly located close to the ventricular system. In these tracts, the occurrence of some WMH was already common at ages <50 years, followed by a (mostly) linear increase of median log10-transformed WMH volume over time with higher absolute volumes for women compared to men. A second curve pattern concerned tracts (superior longitudinal fasciculus (SLF), inferior longitudinal fasciculus (ILF), corticospinal tract and uncinate fasciculus) where WMH were uncommon or even largely absent before the age of 60–65 years, followed by an accelerated WMH burden above that age, with higher volumes by age in women than men. Overall, these tracts are located further away from the ventricles and cover deep/subcortical white matter areas. The third curve pattern concerned tracts (superior longitudinal fasciculus temporal part, cingulum cingulate gyrus, cingulum hippocampus) in which WMH were uncommon at any age for both sexes. In these tracts, the median curve never left the baseline. These tracts are located at para-sagittal/medial temporal areas.

## 4. Discussion

This study shows that total WMH volume increased exponentially with aging for both men and women, with doubling of median volume approximately every 10 years. The key feature of WMH volume that is highlighted by this study is its marked interindividual variance, which is substantial compared to the age effect and this variance also increased exponentially with aging. Women showed higher WMH volumes compared to men from 40 years onwards with a more rapid increase at higher ages. In tract-based analyses, we identified three different patterns with aging: tracts that followed the same curves as total WMH volume, tracts where WMH accumulation started later, approximately at the age of 60–65 years, and accelerated at higher ages, and tracts where WMH were rare even at advanced ages.

One of the primary challenges in the pooling of datasets from multiple studies is to reliably harmonize data and to identify heterogeneity that can potentially bias the results. The included studies differed in recruitment strategies, response rates and in- and exclusion criteria, most evidently reflected in differences in included age ranges (see Figure 1, Table S1). Furthermore, there were differences in image acquisition (e.g., field strength, vendor, MR sequences) and processing. The combination of disjointed age ranges and cohort differences beyond imaging acquisition and processing precluded the use of image harmonization methods such as ComBat (Orlhac et al., 2022) to correct for heterogeneity in image acquisition. Instead, we chose to register WMH masks to MNI-152 space to correct for differences in head and brain size, align world coordinates, enable the removal of voxels located outside the white matter as an extra cross-cohort quality control step and to facilitate analyses across participants. Despite heterogeneity among cohorts, the harmonized data were sufficiently homogeneous to justify merging data of all cohorts. This is supported by our supplemental analyses in the two largest cohorts (RS and HCHS, see Section 3.2) and our second sensitivity analysis considering image processing (Figure S7, Table S4). These analyses demonstrate that the cohort-specific effects and the impact of image processing method both had modest effects on median estimates, which was only a fraction of the interindividual variation observed both within and across cohorts.

We targeted the general dementia-free population. Because we aimed to create normative data on WMH volumes that are representative of the general aging population, rather than focusing on a select group of healthy individuals, we did not additionally exclude individuals with mild cognitive impairment, a history of stroke, other neurological disorder (e.g. migraine), manifest cardiovascular disease, or unfavorable vascular risk profiles, even though these may clearly affect WMH burden. The issue is that excluding these common medical disorders or risk factors that are highly prevalent in the population reduces generalizability. Our sensitivity analyses show that exclusion of those with a history of stroke from all cohorts leads to slightly lower WMH volume estimates, as expected, however, the magnitude of this effect (1–2 % reduction of the median, particularly above the age of 65) is again a mere fraction of the overall interindividual variation.

Prior studies have shown that WMH volume increases non-linearly with aging (Fatemi et al., 2018; Garnier-Crussard et al., 2020; Habes et al., 2018b, 2016; Huang et al., 2018; King et al., 2014; Lohner et al., 2022; Yang et al., 2016), of which some suggest a faster acceleration at higher ages (Garnier-Crussard et al., 2020; Habes et al., 2016; Yang et al., 2016), but the age at which this acceleration occurred was inconsistent. Our multicenter study shows that the WMH volume increase follows a remarkably stable, exponential pattern with aging, with a slight acceleration in females over the age of 65–70 years. There is mounting evidence that burden of WMH is higher in older females, compared to males (Fatemi et al., 2018; Habes et al., 2016; Lohner et al., 2022; Nyquist et al., 2015). Postmenopausal status has been suggested as an important factor explaining differences in WMH volume and progression between sexes (Lohner et al., 2022). Of note, sex differences in our study were already detectable at the age of 40, typically suggested as a premenopausal age in women. There are other reproductive factors that are suggested to play a role in sex differences in cardiovascular disease risk (Ardissino et al., 2023), but these have not been studied in the specific context of SVD. In terms of effect sizes, Fatemi et al. reported differences in WMH volume between sexes were equivalent to seven years of aging (Fatemi et al., 2018). Our results suggest that these differences between sexes are smaller, equivalent to an aging effect of two to five years. In line with Fatemi et al., the age effect in our study is substantially larger (i.e., doubling of median WMH volumes every 10 years of age for both sexes) than the sex-effect alone.

The key feature of WMH volume that is highlighted by this study is its marked interindividual heterogeneity. Table 2 shows that from the age of 40–85, the median WMH volume increases 20–26-fold. Moreover, within each age category, individuals at the 90th percentile typically have a 15-fold higher volume than those at the 10th percentile, clearly with even more extreme differences between the 5th and 95th percentiles and with some individuals having no WMH even above the age of 65. Although most prior studies do not explicitly report variance of WMH volumes at a given age, scatterplots of WMH volume versus age (Fatemi et al., 2018; Habes et al., 2016; Lohner et al., 2022) and reported standard deviations and interquartile ranges of median WMH volume for different age ranges (Huang et al., 2018; King et al., 2014) show comparable large variance. This large normal variance is remarkable and warrants further investigation, also beyond the role of conventional vascular risk factors as explanatory variables. Together, in our view, these findings caution against using terms as “brain age” based on normative WMH data, as a subject at the 90th percentile, generally considered still within the range of normal variance, would be classified as having a WMH age 15 years above her or his chronological age. This will likely sound less comforting than: “one in ten individuals of your age has similar scan findings.”

To the best of our knowledge, this is the first study to describe three differential patterns for tract-specific WMH volumes with aging. Several other studies compared periventricular WMH with deep/subcortical WMH (Habes et al., 2016; Huang et al., 2018; Nyquist et al., 2015; Yang et al., 2016). Although most studies report an increase of WMH with aging in both areas, the age at which accumulation of WMH starts and the rate of increase yield conflicting results, likely also attributable to different classifications of these subregions (Yang et al., 2016), which is suggested to be arbitrary (DeCarli et al., 2005). Our results fit with the observations that periventricular WMH accumulation starts early in life and that the rate of age-dependent increase in periventricular WMH volume resembles that of total WMH volume (Huang et al., 2018). Two other studies, within the same population, indicated that accumulation of WMH in frontal regions starts earlier, but with a slower rate of increase compared to posterior regions (Habes et al., 2018b, 2018a). Tracts studied in the current study are generally large WM-tracts, passing both anterior and posterior regions, but patterns of more frontally located tracts (e.g. ATR, curve pattern 1) and more posteriorly located tracts (e.g. SLF or ILF, curve pattern 2) largely converge with these prior findings, with the exception of the FMaj. The identification of three differential curve patterns for tract-specific WMH volumes is relevant for our understanding of both causes and consequences of WMH. For instance, tracts that appeared vulnerable to early WMH accumulation (curve pattern 1) were also recently identified as strategic to cross-domain cognitive impairment in a large memory-clinic setting (Coenen et al., 2023). Furthermore, some tracts may be either susceptible or resilient to WMH accumulation and distinct patterns might be related to specific underlying etiologies (e.g., arteriosclerosis or amyloid pathology) (Biesbroek et al., 2024), but this needs further exploration. Possibly, regions seldom affected by WMH may hold particular relevance for pathology or adverse clinical outcomes.

When interpreting WMH volumes in individuals, clinicians should be aware of the aging effect, sex differences and large interindividual variation reported herein. The normative data enable a quantitative approach to interpreting WMH volumes that places an individual on a specific percentile taking into account their age and sex. This quantitative and individualized approach to WMH volume can be valuable in various clinical settings, such as in memory clinics, or when an asymptomatic patient presents with covert WMH on brain MRI. It helps to contextualize the WMH volume of an individual patient and thereby enhances the ability to inform patients effectively. To facilitate this approach, a freely available online tool for calculating individual total and regional WMH volumes and corresponding percentile scores will be made available (at https://metavcimap.org/lsm-viewer/#). Also, Figure S3 shows examples of WMH maps for visual estimation of an individual’s WMH volume. We chose to report percentiles, because their interpretation is intuitive which facilitates effective communication of test results with patients. As a cautionary note, confidence intervals for ages <40 and >85 years were relatively wide and individual percentile scores may be less precise. Furthermore, although our reported percentiles curves are primarily driven by aging related WMH of presumed vascular origin (Pantoni, 2010), percentiles for individual patients should always be interpreted within the clinical context. An extensive WMH burden, particularly in case of unusual patterns such as marked asymmetry, may very well be caused by non-vascular etiologies in individual patients (Wang et al., 2022). Future studies should determine if there are specific thresholds of WMH burden above which people may particularly benefit from proposed strategies to prevent adverse clinical outcomes (Smith et al., 2017a; Wardlaw et al., 2022). Finally, the use of quantitative WMH volumes requires image processing, i.e., lesion delineation and correction for head size, which is achieved by registration to standard space in our protocol, that is usually not yet part of routine imaging protocols. The increasing availability of validated and reliable automated segmentation methods (Kuijf et al., 2019) and MRI brain image registration kits, such as RegLSM (Biesbroek et al., 2019), combined with the availability of normative data, will hopefully lead to the integration of these technologies into (widely available) radiology software in the near future.

Strengths of our study are the multicenter setting with marked geographic diversity, spanning seven countries and four continents. There was a balanced representation of both sexes and large sample sizes across ages, particularly over the 40–85-year intervals, resulting in high model confidence for this age range. Through quantile regression (Koenker and Bassett, 1978) we were able to take into account non-linear age terms, which enhanced the precision of created centile curves (Huang et al., 2018; Lohner et al., 2022; Yang et al., 2016). These strengths enabled to provide precise estimates of the normal variance and the rate of increase of WMH across the adult lifespan. Finally, registration of all individual WMH-maps to standard space enabled a uniform atlas-based approach to gain insight into centile curve patterns of tract-specific WMH volumes with aging.

There are also limitations to our study. Despite the wide geographic coverage of the included cohorts, the data reflect a predominantly white population and there is a developed-world bias, as in most MR studies. The majority of non-White individuals included in our dataset comprised individuals of Asian race and Figure S4 demonstrated that centile curves were comparable between CU-RISK and the pooled dataset. The ethnic diversity within the remaining individuals of non-white race and ethnicity was too extensive to support stratified analyses for other races and ethnicities. Therefore, applying our normative data to populations composed entirely of non-white races and ethnicities should be done with caution, as prior literature suggests that WMH burden might vary across races and ethnicities (Sudre et al., 2018). Ideally, the normative curves should be validated for other races and ethnicities, which requires population cohorts that target a more diverse population. Furthermore, as discussed previously, there was heterogeneity in image acquisition and processing. Although the effects on resultant normative data are expected to be small in the light of the observed interindividual variance, individuals with a very low WMH volume might have been disproportionally affected by this heterogeneity. Furthermore, we cannot rule out the introduction of small errors in WMH segmentation or that some individuals had WMH due to other causes than aging related WMH of presumed vascular origin. Next, in participants with severe atrophy or an innate smaller head and therefore brain, a more extensive transformation is needed, which may have affected the precision of volumetric WMH measures in these subjects. As a more general limitation of population studies, healthy self-selection bias (i.e., participants who volunteer to take part in research tend to be healthier) may have influenced the results, especially at older ages.

## 5. Summary and conclusions

WMH volume increases exponentially with age, doubling every 10 years in both sexes, with a marked interindividual variance at all ages. Compared to males, WMH volumes in females were higher from the age of 40 onwards. There were notable differences in tract-specific WMH volumes with aging, some tracts following the pattern of total WMH volume across ages, whereas in other tracts WMH only occurred at higher ages. The age- and sex-specific normative data provided by this study permit a quantitative and individualized approach to interpreting WMH volumes. We recommend to provide such estimates in terms of percentiles, as terms like brain age might be less informative given the observed variance.

## Supplementary Material

supplementary material

## Figures and Tables

**Table 1:** Baseline characteristics, stratified by sex.

	Females (n=7762)	Males (n=7114)	Pooled cohort (n=14876)

Age in years. mean (SD)	63.7 (12.1)	64.1 (11.7)	63.9 (11.9)
Race^†^			
- White. n (%)	6849 (89.1)	6165 (87.5)	13014 (88.3)
- Black. n (%)	232 (3.0)	101 (1.4)	333 (2.3)
- Asian. n (%)	477 (6.2)	691 (9.8)	1168 (7.9)
- Other. n (%)	128 (1.7)	92 (1.3)	220 (1.5)
Educational level^#†^			
- Lower. n (%)	1552 (20.4)	1421 (20.3)	2973 (20.3)
- Intermediate. n (%)	2892 (37.9)	2134 (30.5)	5026 (34.4)
- Higher. n (%)	3179 (41.7)	3438 (49.2)	6617 (45.3)
Hypertension. n (%)^†^	4130 (53.7)	4298 (61.0)	8428 (57.2)
Diabetes mellitus. n (%)^†^	1082 (14.2)	1255 (17.9)	2337 (16.0)
Hypercholesterolemia. n (%)^†^	2910 (38.1)	2667 (38.0)	5577 (38.1)
Smoking habit^†^			
- never. n (%)	4958 (66.6)	4291 (62.0)	9249 (64.4)
- current. n (%)	876 (11.8)	888 (12.8)	1764 (12.3)
- past. n (%)	1607 (21.6)	1744 (25.2)	3351 (23.3)
BMI (kg/m^2^). mean (SD) ^‡^	26.9 (5.0)	27.3 (4.1)	27.1 (4.6)
Atrial fibrillation. n (%)*	125 (2.9)	206 (4.8)	331 (3.8)
Cardiovascular disease. n (%)*	417 (8.0)	587 (11.5)	1004 (9.7)
History of stroke. n (%)^†^	107 (1.4)	126 (1.8)	233 (1.6)
WMH volume (mL). median (IQR)	2.2 (0.9–5.1)	1.8 (0.8–4.4)	2.0 (0.8–4.8)

# Educational level: lower: less than high school completion, intermediate: high school completion, higher: all education beyond high school completion.

† Missing in <5%

‡ Missing in 5–10%

* Missing in 10–50%.

Abbreviations: BMI, Body Mass Index; IQR, interquartile range; kg, kilograms; m, meters; mL, milliliter; SD, standard deviation; WMH, white matter hyperintensities.

**Table 2:** Normative values for absolute total white matter hyperintensity volume, by age and sex.

	Percentiles						
	p5	p10	p25	p50	p75	p90	p95

**Age (years)**	**Females**						
	
40	0.03	0.07	0.16	0.39	0.83	1.41	2.02
45	0.06	0.11	0.26	0.57	1.13	1.84	2.58
50	0.09	0.18	0.40	0.83	1.53	2.47	3.45
55	0.14	0.27	0.59	1.16	2.09	3.44	4.85
60	0.21	0.38	0.83	1.59	2.86	5.02	7.26
65	0.31	0.56	1.17	2.22	4.18	7.96	11.98
70	0.49	0.86	1.70	3.32	6.77	13.91	21.73
75	0.76	1.32	2.50	5.00	10.80	23.03	36.25
80	1.13	1.89	3.61	7.29	15.59	32.86	49.78
85	1.61	2.56	5.14	10.31	20.73	41.52	58.38
	
**Age (years)**	**Males**						
	
40	0.03	0.06	0.14	0.35	0.74	1.28	1.86
45	0.05	0.10	0.22	0.50	0.95	1.61	2.34
50	0.09	0.15	0.34	0.71	1.27	2.12	3.11
55	0.13	0.23	0.52	1.00	1.74	2.95	4.40
60	0.20	0.34	0.76	1.40	2.48	4.37	6.69
65	0.26	0.48	1.06	1.98	3.70	6.86	10.77
70	0.31	0.63	1.38	2.87	5.72	11.12	17.83
75	0.36	0.82	1.78	4.08	8.60	17.24	27.38
80	0.48	1.15	2.37	5.52	12.00	24.28	36.53
85	0.69	1.69	3.26	7.17	15.78	31.66	43.61

Percentiles were derived from quantile regression models for the specific ages shown. Volumes are absolute normalized white matter hyperintensity volumes in MNI-152 space, shown in milliliter.

Abbreviations: p, percentile.
