# Supplementary material for: Cerebral white matter hyperintensity volumes: Normative age- and sex-specific values from 15 population-based cohorts comprising 14,876 individuals
Source: Neurobiol Aging. Author manuscript; Available in PMC 2025 May 19. (PMC12087372; doi:10.1016/j.neurobiolaging.2024.11.006)
Supplement: supplementary material [file NIHMS2077596-supplement-supplementary_material.pdf]

Appendix A. Supplemental Material

Supplementary methods

1. Table S1: Cohort-specific details
2. Harmonization methods
3. MRI acquisition
4. Image processing
5. Figure S1: Flowchart of participant selection
6. Figure S2: Lesion prevalence maps stratified by sex
7. Figure S3: Examples of white matter hyperintensity distributions corresponding to log10-transformed volumes

Supplementary results

1. Table S2: Baseline, stratified by cohort
2. 5-year age strata and inter- and intracohort observations
3. Figure S4: Cohort-specific distribution of log10-transformed white matter hyperintensity volume versus age
4. Figure S5: Centile curves for absolute white matter hyperintensity volumes, stratified by sex
5. Sensitivity analyses: Figures S6, S7; Tables S3, S4
6. Figure S8: Centile curves and absolute white matter hyperintensity volumes for the three identified curve patterns of white matter tract-specific volumes

Table S1: Cohort-specific details

Cohort	Study design	Population	Recruitment strategy	Recruitment period	Age criterium	Other main inclusion criteria	Main exclusion criteria	Response rate study	Sample size at baseline	Participants invited for brain MRI; response rate MRI	Population/ datapoints used in current study
Austrian Stroke Prevention Study - Original (ASPS) (Schmidt et al., 1994)	prospective single-center population based	population of Graz, Austria	- a random population sample, via the official community register, received a written invitation to participate<br>- balanced distribution based on sex and 5-year age groups	1991-1994	50-75 years	none	- history of neuropsychiatric disease, including stroke and dementia<br>- abnormal neurologic examination at structural clinical examination	34% interested and eligible	1998 individuals (enrollment stopped after inclusion of 1998 individuals)	every 4th participant; 92%	baseline MRI and corresponding clinical data. Follow-up MRI and data were used in case of missing or bad quality FLAIR, and no overlap with ASPSF
Austrian Stroke Prevention Family Study (ASPSF) (Seiler et al., 2014)	prospective single-center offspring study	population of Graz, Austria	- study participants of ASPS and their first-grade relatives were asked to participate	2006-2013	50-75 years	none	- history of neuropsychiatric disease, including stroke and dementia<br>- abnormal neurologic examination at structural clinical examination	N/A	381 individuals	all enrolled were invited; 93%	baseline MRI and corresponding clinical data
Alzheimer's Disease UC Davis Diversity Cohort (AUCD) (Hinton et al., 2010)	prospective two-center population based	population of California, served by the University of California, Davis Alzheimer's Disease Center	-community outreach program<br>- Recruitment goals were focused on 1) a cognitively heterogeneous sample 2) of 1/3 Hispanic, 1/3 African American and 1/3 non-Hispanic Caucasian decent	2002–2007	≥ 60 years	fluent in English or Spanish	- unstable major medical illness<br>- major primary psychiatric disorder<br>- substance abuse or dependence in the last 5 years	N/A	1357 individuals completed cognitive screening with SENAS at baseline	all enrolled in the longitudinal cohort were invited; 86%	a random sample of screened individuals with no significant impairment on one of the SENAS scales (measuring episodic memory, language/ semantic memory, spatial ability, abstract reasoning, and attention span)

Calgary Normative Study (CNS) (McCreary et al., 2020)	prospective single-center population based	population of Calgary, Canada	- via posters and word-of-mouth<br>-balanced distribution based on sex and 10-year age groups	inclusion started 2013, ongoing	≥ 18 years	none	- history of neuropsychiatric disease, including stroke and dementia<br>- contra-indications for MRI	N/A	ongoing	all enrolled; 100%	baseline MRI and corresponding clinical data
The Chinese University of Hong Kong - Risk Index for Subclinical brain lesions in Hong Kong Study (CU-RISK) (Wong et al., 2015)	prospective single-center population based	population of Hong Kong SAR, China	- advertisements in local community-centers and word-of-mouth	2011-2015	≥ 65 years	- functional independence as defined by a score of 20 on the 20-point Barthel Index and < 2 on the Lawton's Instrumental of Daily Living Scale (IADL)<br>- Cantonese-speaking<br>- sufficient sensorimotor and language competency for cognitive testing	- history of neuropsychiatric disease, including stroke, TIA and dementia<br>- evidence of brain tumors, large cerebral infarcts (i.e., infarcts ≥20 mm in diameter), cortical infarcts, or hydrocephalus on MRI	N/A	830 individuals	all enrolled; 100%	baseline MRI and corresponding clinical data
Framingham Heart Study-Offspring cohort (FHS_ Gen2) (Feinleib et al., 1975)	prospective single-center offspring study	population of Framingham, USA	- biological children and their spouses of the Original FHS-cohort members were asked to participate	inclusion started 1971; health examinations ± every 4 years	12-60 years, at baseline	none	- history of neuropsychiatric disease, including stroke and dementia	Ca. 80%	5124 individuals attended first round of gen II	all attendees of exam 8 (n=3021) were invited; 51%	exam 8 and corresponding MRI (2005-2008)
Framingham Heart Study- third generation cohort (FHS_ Gen3) (Splansky et al., 2007)	prospective single-center offspring study	population of Framingham, USA	-adults with at least one parent in the Offspring Cohort received invitation letters<br>-priority given to extended families	first round 2002-2005	19-72 years, at baseline	none	- history of neuropsychiatric disease, including stroke and dementia	62,5% interested and eligible	4095 individuals attended first round of gen III	all attendees of exam 2 (n=3411) were invited; 59%	exam 2 and corresponding MRI (2009-2011)

Framingham Heart Study-OMNI-I cohort (FHS_ OMNI1) (Quan et al., 1997)	prospective single-center population based	population of Framingham, USA	Framingham residents who self-identified as members of a minority group (African American, Hispanic, Asian, Indian, Pacific Islander and Native American descent)	inclusion started 1994	40-74	none	- history of neuropsychiatric disease, including stroke and dementia	N/A	506 participants attended first round of OMNI-I	all attendees of exam 3 (n=298) were invited; 48%	exam 3 and corresponding MRI (2005-2008)
Hamburg City Health Study (HCHS) (Jagodzinski et al., 2020)	prospective single-center population based	population of Hamburg, Germany	- a random sample from the official inhabitant data file received written invitation letters -divided into six age and gender strata	2016-ongoing.	45–74 years	none	none	N/A	enrollment ongoing at this time. First round (2016-2018) included 10.000 individuals	subgroup of 2,657 from 10.000 participants selected for brain MRI	baseline MRI and corresponding clinical data
Lothian Birth Cohort 1921 (LBC1921) (Deary et al., 2004; Ritchie et al., 2018)	observational longitudinal, population based	population of the Lothian area, Scotland	- individuals born in 1921 who now live in the Lothian area of Scotland, identified using the Community Health Index, received written invitation letters (including one reminder), followed by media advertisement	1999-2001	birth-year 1921	- availability of test results of the Scottish Mental Survey of 1932	none	49,1% of all invited individuals were included at wave 1	550 individuals were included at wave 1	brain imaging was included in wave 5; all enrolled invited; 90%	wave 5 (2013)
Lothian Birth Cohort 1936 (LBC1936) (Deary et al., 2007)	observational longitudinal, population based	population of the Lothian area, Scotland	- individuals born in 1936 who now live in the Lothian area of Scotland, identified using the Community Health Index, received written invitation letters (including one reminder), followed by media advertisement	2004-2007	birth-year 1936	- availability of test results of the Scottish Mental Survey of 1947	none	29,6% of all invited individuals were included at wave 1	1091 individuals were included at wave 1	brain imaging was included in wave 2; all enrolled invited; 84%	wave 2 (2007-2010)

Sydney Memory and Ageing Study (MAS)(Sachdev et al., 2010)	prospective single-center population based	population of Sydney, Australia	- a random population sample through the electoral roll from two federal government areas, participants received invitation letters	2005-2007	70-90	- availability of an informant who had to have at least weekly contact of not less than one hour with the participant	- history of neuropsychiatric disease, including dementia, progressive malignancy or psychotic symptoms<br>- medical or psychological conditions that may have prevented them from completing assessments<br>- MMSE score of < 24	19,9% interested	1037 individuals attended baseline assessment	all enrolled were invited; 53%	wave 1 (baseline)
Older Australian Twins Study (OATS)(Sachdev et al., 2009)	prospective single-center	twins and their siblings, New South Wales, Victoria and Queensland, Australia	- twins were contacted through the Australian Twin registry, media release and newspaper advertisement.	2006-2012	>65 years	- having a consenting co-twin<br>- having completed some education in English<br>- at least low average intelligence (IQ ≥ 80).	- diagnosis of malignancy or other life-threatening illness or acute psychosis- inadequate English to participate in assessments	N/A	623 individuals (600 twins, 23 siblings) attended baseline assessment	all enrolled were invited; 66%	wave 1 (baseline)
Rotterdam Study (RS)(Hofman et al., 2015; Ikram et al., 2015)	prospective single-center population based	population of Ommoord, Rotterdam, The Netherlands	- a random population sample, participants received invitation letters	RS-I: 1990-1993<br>RS-II: 2000<br>RS-III: 2006	RS-I and RS-II: ≥55 years<br>RS III: ≥ 45 years	none	none	combination of RS-I, RS-II and RS-III at baseline: 72%	combination of RS-I, RS-II and RS-III at baseline: 14,926 individuals	all enrolled were invited; RS-I-5: 81% RS-II-3: 80% RS-III-2: 75%	combination of waves RS-I-5, RS-II-3, RS-III-2; all with availability of MRI (protocol > 2005) and corresponding clinical data

Southall and Brent Revisited Study (SABRE) (Tillin et al., 2012)	follow-up population based cohort study	population of West London, UK	- for the original Southall and Brent cohort studies, participants were recruited from either their workplaces (20%) or randomly selected from general practice registers (80%).<br>- at wave 3, partners of each index participant were invited and new recruitment of African Caribbean participants was carried out by clinic staff who went into local communities.	original cohort studies: 1988-1991<br>SABRE follow-up study: 2008-2012<br>Wave 3: 2014-2018	original cohort studies: 40-69 years<br>SABRE follow-up study:<br><br>Wave 3: Index: 65-90 years, new participants: 37-90 years	None	None	original cohort studies at baseline (combined): 63%<br>Wave 3: N/A	original cohort studies at baseline (combined): 4972 individuals<br>SABRE: 1438 individuals<br>Wave 3: 1000 individuals (Index: 654, new partners: 249 and new African Caribbean: 97)	all enrolled were invited; 78%	wave 3 (2014-2018)

Harmonization methods

Given the heterogeneity in data collection methods employed by the different cohorts, the data required harmonization steps. Below, a more detailed description and justification of the variable recoding is given.

Variables for ***Sex, Body Mass Index, History of stroke and Atrial fibrillation*** could be unambiguously recoded to confirm the definitions specified below. Participants of ASPS, ASPSF, FHS, CNS and CU-RISK were assumed to have no history of stroke, as a history of stroke was one of the original exclusion criteria of the cohort.

Schematic representation of variables unambiguously recoded

**Variable**	**Definition**	**Categories/ units**
Sex	Sex assigned at birth	Male / female
BMI	Body Mass Index	kg/m^2
History of stroke	A history of stroke	Yes / no
Atrial fibrillation	Diagnosis of atrial fibrillation	Yes/ no

Age
Age was defined as the chronological age in years at time of imaging. For participants of the SABRE cohort, only the birth year was provided. As all participants were evaluated between 2014 and 2016, the age was approximated by the difference between birth year and 2015.

Race and ethnicity
Based on availability of data, race and ethnicity was recoded into four categories: White, Black, Asian and Other. The categories included in the "other" group were too small to make separate categories, however, to be as specific as possible, details on the races and ethnicities included in this group are provided below. For the LBC1936, LBC1921, ASPS, ASPSF and HCHS all individuals were Caucasian White and were labeled as White. For the RS, 97% of individuals were assumed to be White.

Schematic representation of the recoding of race

**Cohort**	**Asian**	**Black**	**White**	**Other**
CNS		Black	White	Hispanic, First Nations
MAS/ OATS	Asian	African	Caucasian	Indigenous Australian, Torres Strait Islander, Pacific Islander, Mixed
SABRE	South-East Asian	African Caribbean	European	Other
AUCD	Asian	African American	White	Pacific Islander, Filipino, Hispanic, Other
FHS	Asian	Black	White	Hispanic or Latino, Native Hawaiian/Pacific Islander, American Indian/Alaskan Native, Asian Indian/Pacific Islander, Multiracial

Level of education
Educational level and years of education were harmonized into three levels:
1. Lower (less than high school completion)
2. Intermediate (high school completion)
3. Higher (all education beyond high school completion)
For cohorts with years of education provided, we harmonized data conform the STROKOG-consortium as described by Lo et al. (2019)(Lo et al., 2019) and interpretation of these levels by Weaver et al. (2021)(Weaver et al., 2021). Years of education was converted to education level as shown below, based on structure of the local educational system. To match with data from the RS and FHS, STROKOG 3 and 4 were merged to level 3: "higher" educational level.

STROKOG 1: less than high school completion → Lower
STROKOG 2: high school completion → Intermediate
STROKOG 3: technical or college diploma → Higher
STROKOG 4: university degree or above → Higher

Schematic representation of the recoding strategy for years of education into educational level (1)

**Country**	**Cohorts**	**Birthyear**	**Years of education****			**Source/ comments**
			**Lower**	**Intermediate**	**Higher**	
Australia	MAS<br>OATS	Any	<13	13	>14	Lo et al. (2019)(Lo et al., 2019)
Austria	ASPS<br>ASPSF	Any	<10	10-12	>13	Federal ministry of labour and economics
Canada	CNS	Any	<13	13-16	>16	Government of Canada (2022)
England*	SABRE	<1935	<15	15-20	>20	Weaver et al. (2021)(Weaver et al., 2021)
		>1934	<16	16-20	>20	
Germany	HCHS	Any	<10	10-12	>13	Free and Hanseatic City of Hamburg.
Hong Kong	CU-RISK	Any	<14	14	>15	Weaver et al. (2021)(Weaver et al., 2021)
Scotland	LBC1921<br>LBC1936	Any	<11	11-16	>16	Weaver et al. (2021)(Weaver et al., 2021)
USA	AUCD	Any	<13	13	>14	Lo et al. (2019)(Lo et al., 2019)

*Because of reform of the English educational system in 1947, educational level is classified differently for participants born after 1934.
** Years of education was self-reported.

Schematic representation of the recoding strategy for educational level (2)

**Country**	**Cohorts**	**Level of education**		
		**Lower**	**Intermediate**	**Higher**
The Netherlands	RS	1. Primary education	2. Lower/ intermediate general education OR lower vocational education<br>3. Intermediate vocational education OR higher general education	4. Higher vocational education or university
USA	FHS	1. Less than high school completion	2. High school completion	3. Some college<br>4. College graduate

Smoking status

Smoking status was harmonized into 3 categories: current, past and never. HCHS defined only two categories of smoking habits, therefore, participants who did not currently smoke were assumed to have never smoked.

Hypertension

No universal definition could be used. Instead, the following local definitions were used to create a category with binary outcome (yes/no).

ASPS/ ASPSF
Three blood pressure measurements >160/95mmHg or
the use of antihypertensive medication.

AUCD
Self-reported history of hypertension.

CNS
A self-reported history of hypertension or
the self-reported use of antihypertensive medication.

CU-RISK
Blood pressure measurements >140/90mmHg or
the use of antihypertensive medication.

FHS
Systolic blood pressure ≥140mmHg or diastolic blood pressure ≥90mmHg or
the use of antihypertensive medication

HCHS
A blood pressure measurement >140/90mmHg,
the use of antihypertensive medication or
a self-reported history of hypertension.

LBC1921/ LBC1936
Self-reported history of ever being treated for high blood pressure

MAS/OATS
Repeated blood pressure measurements with a mean >140/90mmHg or
the use of antihypertensive medication.

RS
Two blood pressure measurements with a mean systolic blood pressure of >140mmHg or a mean diastolic blood pressure of >90mmHg or
the use of antihypertensive medication.

SABRE
Physician diagnosed hypertension or
the self-reported use of antihypertensive medication.

Diabetes
No universal definition could be used for diabetes. Instead, the following local definitions were used to create a category with binary outcome (yes/no).

ASPS/ ASPSF
A fasting glucose >140mg/dL or
the use of antidiabetic medication.

AUCD
A self-reported history of diabetes.

CNS
A self-reported history of diabetes or
the use of antidiabetic medication.

CU-RISK
A fasting glucose >6,0mmol/L,
HbA1c >5,7% or
the use of antidiabetic medication.

FHS
fasting plasma glucose of ≥126 mg/dL, random plasma glucose ≥140, or
the use of antidiabetic medication.

HCHS
A self-reported history of diabetes or
the use of antidiabetic medication.

LBC1921/LBC1936
A self-reported history of ever being diagnosed with diabetes

MAS/OATS
Physician diagnosed diabetes (not further specified).

RS
A fasting glucose of >7,00mmol/L or
the use of antidiabetic medication.

SABRE
A fasting glucose >7,00mmol/L or
physician diagnosed diabetes (not further specified).

Hypercholesterolemia
None of the cohorts provided blood cholesterol measurements allowing a uniform definition of hypercholesterolemia. Instead, the following local definitions were used to create a category with binary outcome (yes/no).

ASPS/ ASPSF
HDL cholesterol >40mg/dL in men and >50mg/dL in women or
the use of lipid lowering medication.

AUCD
A self-reported history of hypercholesterolemia

CNS
A self-reported history of hypercholesterolemia or
the use of lipid lowering medication.

CU-RISK
Elevated serum lipids (not further specified) or
the use of lipid lowering medication.

FHS
Total cholesterol and HDL levels were provided in mg/dl. The cut-off value of >240 mg/dl for total cholesterol was used to define individuals with hypercholesterolemia.

HCHS
LDL/HDL ratio >3,5 or
the use of lipid lowering medication.

LBC1921/LBC1936
A self-reported history of ever being diagnosed with high cholesterol

MAS/OATS
Physician diagnosed hypercholesterolemia (not further specified).

RS
Total cholesterol >4,14 mmol/l or
the use of lipid lowering medication.

SABRE
Use of lipid lowering medication

Cardiovascular disease
The cohorts provided details on the following forms of cardiovascular disease.

ASPS/ ASPSF
A history of symptoms of acute coronary syndrome or

signs of coronary artery disease and left ventricular hypertrophy on electrocardiogram or echocardiogram.

AUCD
A history of acute coronary artery syndrome or
previous angioplasty or coronary artery bypass graft.

CU-RISK
A history of acute coronary artery syndrome.

FHS
A history of myocardial infarction or
coronary insufficiency

HCHS
A history of myocardial infarction.

LBC1921/LBC1936
A self-reported history of ever being diagnosed with cardiovascular disease, including heart attack, angina, heart valve problems, abnormal heart rhythm or any other heart problem.

MAS/OATS
A history of acute coronary artery syndrome or peripheral arterial occlusive disease.

SABRE
A history of acute coronary artery syndrome or
Previous angioplasty or coronary artery bypass graft.

MRI acquisition

ASPS
MRI was performed on a 1.5T scanner (Gyroscan S 15 and ACS, Philips, Eindhoven, The Netherlands). The protocol included axial T2-weighted sequences (TR 2000 to 2500 msec, TE 30 to 90 msec) and sagittal T1-weighted images (TR 600, TE 30 msec) with slice thickness of 5 mm and a matrix size of 128 x 256 pixels (Schmidt et al., 1999).

ASPSF
MRI was performed on a 3T scanner (TimTrio; Siemens Healthcare, Erlangen, Germany). The protocol included an axial FLAIR sequence (TR 10000 ms, TE 69 ms, TI 2500 ms, slice thickness 3 mm, in-plane resolution 0.86 mm × 0.86 mm) and a 3D-T1 with magnetization preparation (MPRAGE) and whole brain coverage (TR 1900 ms, TE 2.19 ms, TI 900 ms, flip angle 9°, isotropic resolution of 1 mm) (Seiler et al., 2014).

AUCD
All participants underwent a standardized research MRI protocol, as described by Hinton et al. (2010) (Hinton et al., 2010).

CNS
MRI was completed on a 3T scanner (MR750, General Electric Healthcare, Waukesha, Wisconsin, USA) using the vendor-supplied, 12-channel head, neck and spine coil. The MRI protocol included conventional structural 3D-T1 (slice orientation coronal, TR 7 ms, TE 2.5 ms, flip angle 8°, matrix 256x256, resolution 0.94x0.94x1.0, TI 650ms) and FLAIR sequence (slice orientation axial, TR 9000 ms, TE 3.5 ms, Flip angle 90°, matrix 256x256, resolution 0.94x0.94x1.0 mm^3, TI 650 ms) (McCreary et al., 2020).

CU-RISK
MRI was acquired using a 3T scanner (Achieva 3.0 T X-series, Philips Medical System, Best, the

Netherlands). The scan protocol was updated during inclusion of participants. In short, scan protocol 1 (n=787) included a 3D T1 (slice orientation transversal, TR 7.49 ms, TE 3.46 ms, matrix 228 x 227) and 3D FLAIR (slice orientation transversal, TR 8000 ms, TE 328.6 ms, TI 2400 ms, matrix 208 x 208). Scan protocol 2 (n=44) included a 3D T1 (slice orientation transversal, TR 6.49 ms, TE 3.112 ms, matrix 256x256) and 2D FLAIR (slice orientation transversal, TR 11.000 ms, TE 125 ms, TI 2800 ms, matrix 352x234) (Biesbroek et al., 2020).

FHS
All participants were imaged on a 1.5T Magnetom scanner (Siemens Medical, Erlangen, Germany). The scan protocol included 3D T1-weighted coronal spoiled gradient-recalled echo acquisition and T2-FLAIR sequences(Petrea et al., 2024).

HCHS
Images were acquired using a 3T Siemens Skyra MRI scanner (Siemens, Erlangen, Germany). For 3D T1-weighted anatomical images, rapid acquisition gradient-echo sequence (MPRAGE) was used with the following sequence parameters: TR 2500 ms, TE 2.12 ms, 256 axial slices, ST 0.94 mm, and IPR 0.83 × 0.83 mm. 3D T2-weighted FLAIR images were measured with the following sequence parameters: TR 4700 ms, TE 392 ms, TI 1800 ms, 192 axial slices, ST 0.9 mm, and IPR 0.75 × 0.75 mm (Petersen et al., 2022)

LBC1921 and LBC1936
Both cohorts underwent the same structural imaging examination in the Brain Research Imaging Centre, University of Edinburgh (http://www.bric.ed.ac.uk), using a GE Signa Horizon HDx 1.5T clinical scanner (General Electric, Milwaukee, WI). In short, the LBC1921 scan protocol included a 3D T1-weighted fast spoiled gradient echo (slice orientation coronal, TR 9.7ms, TE 4 ms, TI 500 ms, matrix 256x256, slice thickness 1.3mm, in-plane resolution 1 × 1 mm), a T2-weighted (fast spin echo, slice orientation axial, TR 8200 ms, TE 103 ms, matrix 256x256, slice thickness 2.5mm, in-plane resolution 0.94 × 0.94 mm), a T2*-weighted (gradient echo, slice orientation axial, TR 1400 ms, TE 15 ms, matrix 256x256, slice thickness 2.5mm, in-plane resolution 0.94 × 0.94 mm) and a FLAIR (fast spin echo, slice orientation axial, TR 9402 ms, TE 146 ms, TI 2530 ms, matrix 256x256, slice thickness 5mm, in-plane resolution 0.94 × 0.94 mm). The LBC1936 scan protocol included a 3D T1-weighted fast spoiled gradient echo (slice orientation coronal, TR 10ms, TE 4 ms, TI 500 ms, matrix 256x256, slice thickness 1.3mm, in-plane resolution 1 × 1 mm), a T2-weighted (fast spin echo, slice orientation axial, TR 11320 ms, TE 105 ms, matrix 256x256, slice thickness 2mm, in-plane resolution 1 × 1 mm), a T2*-weighted (gradient echo, slice orientation axial, TR 940 ms, TE 15 ms, matrix 256x256, slice thickness 2mm, in-plane resolution 1 × 1 mm) and a FLAIR (fast spin echo, slice orientation axial, TR 9002 ms, TE 147 ms, TI 2200 ms, matrix 256x256, slice thickness 4mm, in-plane resolution 1 × 1 mm) (Ritchie et al., 2018; Wardlaw et al., 2011)

MAS
All participants were imaged on a Philips 3T Achieva Quasar Dual scanner (Philips Medical Systems, Best, The Netherlands) located at the Prince of Wales Medical Research Institute, Sydney. The protocol included a 3D T1 (turbo field echo, slice orientation coronal, TR 6.39 ms, TE 2.9 ms, flip angle 8◦, matrix 256×256, FOV 256×256×190 mm3, and slice thickness 1 mm with no gap between; yielding 1×1×1 mm3 isotropic voxels) and a T2-weighted FLAIR sequence (slice orientation coronal, TR 10000 ms, TE 110 ms, TI 2800 ms, matrix size 512×512, slice thickness 3.5 mm with no gap between slices, yielding spatial resolution of 0.488×0.488×3.5 mm3/voxel (Sachdev et al., 2010).

OATS
Brain imaging is performed on a 1.5T scanner. Siemens scanners with similar year of manufacture and upgrade, are being used in two centers (Melbourne and Brisbane), and a Philips scanner is being used in the third center (Sydney). Matching acquisition protocols are being used in the three centers, with standardization of in-plane resolution and slice thickness. A 3D phantom is used to detect variation across scanners (for correction of geometric distortion), and five volunteers are to be scanned on all three scanners for reliability measures. The standardized protocol is as follows: in-plane resolution 1×1 mm with slice thickness of 1.5 mm T1-weighted contiguous coronal sections through whole brain (T1- TFE sequence and 3D acquisition); T2-weighted FLAIR, orientation axial, slice thickness 3.0mm (Sachdev et al., 2009)

RS
All participants were imaged on a 1.5T MRI unit (General Electric Healthcare, Milwaukee, USA, software version 11x). The protocol included a 3D T1 (gradient-recalled echo, TR 13.8 ms, TE 2.8ms, TI 400ms, flip angle 20◦, matrix 416x256, FOV 25 cm2, slice thickness 1.6 mm) and 2D FLAIR (fast spin echo, TR 8000ms, TE 120 ms, flip angle 90-180◦, slice thickness 2.5mm) (Ikram et al., 2015)

SABRE
MRI images were acquired at a single site on a 3T Philips Achieva scanner, including a sagittal 3D-T1 (inversion-prepared gradient echo, TR 6.9 ms; TE 3.1 ms) and a sagittal 3D FLAIR (TR 4800 ms, TE 125 ms, TI 1650 ms) (Sudre et al., 2018)

Image processing

White matter hyperintensity segmentations

FLAIR images were available for all cohorts. For the ASPS and 15 individuals of the ASPSF, white matter hyperintensities (WMH) segmentations were performed in Utrecht, using a previously validated technique (Camarasa R et al., 2018; Kuijf et al., 2019). Details on this segmentation process can be found in the flowchart (in preparation). WMH segmentations of the remaining cohorts/ individuals were provided by participating centers. For 365 individuals of the ASPSF, WMH were manually segmented, using a custom written IDL program (Exelis Visual Information Solutions, USA). Lesion areas were segmented by combined region growing and local thresholding following manual selection (Seiler et al., 2014). For AUCD and FHS**,** WMH segmentation is performed on a combination of FLAIR and 3D T1 images using a modified Bayesian probability structure based on a previously published method of histogram fitting (DeCarli et al., 1999). Prior probability maps for WMH were created from more than 700 individuals with semi-automatic detection of WMH followed by manual editing. Likelihood estimates of the native image are calculated through histogram segmentation and thresholding. All segmentation is initially performed in standard space resulting in probability likelihood values of WMH at each voxel in the white matter. These probabilities are then thresholded at 3.5 SD above the mean to create a binary WMH mask. Further segmentation is based on a modified Bayesian approach that combines image likelihood estimates, spatial priors, and tissue class constraints. Reliability and biological validity of this method is well-established (Maillard et al., 2022).The CNS used a custom made semi-automated, seed-based 3D region growing tool (Cerebra lesion extraction tool) as described by Gobbi et al. (2012) (Gobbi D et al., 2012) For the CU-RISK WMH segmentations were performed using a semi-automated method. Automated WMH segmentations were first performed on all FLAIR-sequences using a built-in-tool (Shi et al., 2013) of the automatic brain quantification software AccuBrain®(Brain Now Medical Technology Limited, Hong Kong SAR). To enable manual correction of the WMH segmentations, the 3D-FLAIR sequences (n=787) and the corresponding WMH lesion masks were down sampled to transversal slices with a slice thickness of 5 mm. Down sampling was not required for 2D-FLAIR sequences (n=44). All 830 automated WMH segmentations were subsequently checked and manually corrected by experienced raters (JMB and BYKL) (Biesbroek et al., 2020). For the FHS**,** all MRI were transferred to the University of California–Davis Medical Center for centralized reading. The segmentation and quantification of WMH was performed using a semi-automated procedure that has been previously described (DeCarli et al., 2005). The HCHS used a Brain Intensity AbNormality Classification Algorithm (BIANCA)(Griffanti et al., 2016) implemented in FSL. The resulting segmentation masks underwent visual quality control (Petersen et al., 2020). For the LBC1921 and LBC1936, WMH were quantified semi-automatically with MCMxxxVI (Valdés Hernández et al., 2010) by fusing T2*W and FLAIR volumes. False-positive lesions in the insular cortex, cingulate gyrus, anterior temporal cortex and around the floor of the third ventricle were removed manually (Ritchie et al., 2018). For the MAS and OATS, automated WMH detection and classification is performed using in-house software. Details are described at Wen and Sachdev (2004) (Wen and Sachdev, 2004) and Wen et al. (2009)(Wen et al., 2009). The RS used a qualitatively and quantitively validated fully automated brain tissue segmentation method, optimized and extended with white matter lesion segmentation. In short, cerebrospinal fluid (CSF), gray matter (GM) and white matter (WM) are segmented by an atlas-based k-nearest neighbor classifier on multi-modal MRI data. This classifier is trained by registering brain atlases to the subject. The resulting GM segmentation is

used to automatically find a white matter lesion threshold in a FLAIR sequence. False positive lesions are removed by ensuring that the lesions are within the white matter (de Boer et al., 2009). For SABRE**,** the BaMoS algorithm(Sudre et al., 2015) was used, in order to automatically segment WMH. This segmentation algorithm models lesions as a Gaussian Mixture Model under multivariate data (T1-w and FLAIR) and automatically determines the number of Gaussian components required to jointly model healthy tissues and abnormal signals. After convergence, the optimized model is used to produce a probabilistic lesion map that is integrated to produce lesion volume measurements. All lesion segmentations passed quality control by visual assessment (Sudre et al., 2018).

Registration to the MNI-152 template

For this study, all WMH maps were registered to the MNI-152 template (Montreal Neurological Institute) with a 1x1x1 mm3 voxel resolution. The MNI-152 standard-space T1-weighted average structural template is derived from 152 structural images, which have been warped and averaged into the common MNI-152 coordinate system after high-dimensional nonlinear registration (Fonov et al., 2011). For the ASPS, ASPSF, CNS, CU-RISK, HCHS, LBC1921 and LBC1936, registrations to standard space were performed using RegLSM (Biesbroek et al., 2019). For ASPS, ASPSF, CNS, LBC1921 and LBC1936 this processing step was performed centrally at the UMC Utrecht. The FLAIR images were first registered to the corresponding T1 image with a linear registration. The T1 image was subsequently transformed to the T1 1-mm MNI-152 template, with a linear registration followed by a non-linear registration. An age-specific MRI template was used as an intermediate step before the final registration to MNI-152 space in order to improve the quality of the registration by providing a better match between the individual and the template (Fonov et al., 2011). The resulting transformations were combined into a single transformation that was subsequently used to transform the corresponding WMH map to the MNI-152 template. The final registration results of all cases were visually checked for accuracy by FASdK. Failed registrations were excluded. For FHS and AUCD, registration of the WMH maps to MNI space was performed using the following method: whole head structural T1 MRI images were processed by in-house pipelines that were described previously (Fletcher, 2014). The first pipeline step produced brain extractions based on convolutional neural net recognition of intracranial cavity followed by human quality control (Fletcher et al., 2021). This was followed by affine and B-spline registration (Rueckert et al., 2006) of the intracranial cavity image to an age-appropriate structural template image (Kochunov et al., 2001) and native-space tissue segmentation into gray matter, white matter and CSF(Fletcher et al., 2012) and WMH with the aid of each subject's coregistered native T1 and FLAIR images (DeCarli et al., 2005). For MAS and OATS, UBO Detector output WMH masks were in a cohort-specific DARTEL space. FSL's FLIRT and FNIRT were used to generate a non-linear transformation from DARTEL to MNI space. The warp was then applied to the WMH masks in DARTEL space to register them to MNI space. For the RS, all T1-weighted images were segmented into supratentorial GM, WM and CSF using a previously described k-nearest neighbor algorithm, which was trained on 6 manually labeled atlases (Vrooman et al., 2007). FSL software(Smith et al., 2004) was used for GM registration. Then, all GM density maps were nonlinearly registered to the standard GM probability template (Good et al., 2001). For this study, the MNI-152 template was chosen. After that, the derived transformation field was applied to WM segmentation images to register all of them to MNI space. SABRE used an affine followed by a non-rigid registration using NiftyReg (Modat et al., 2014, 2010) to register the T1-weighted image to MNI space. Nearest neighbour interpolation was applied to the lesion segmentation map when transforming from native to MNI space using the resulting transformation.

Quality control

To reduce heterogeneity and minimize the effects of possible misclassifications of other lesion types as WMH during the WMH segmentation procedures, voxels located outside the white matter (defined as a probability below 30% according to the MNI probabilistic white matter atlas) removed from all individual WMH maps. To rule out systematic image processing errors, e,g, left-right flips, a random sample of fully processed WMH maps were compared to the original imaging data for each cohort (n=5 per cohort). Furthermore, cohort-specific prevalence maps were visually checked by FASdK and JMB. These quality control steps revealed no data handling or processing errors.

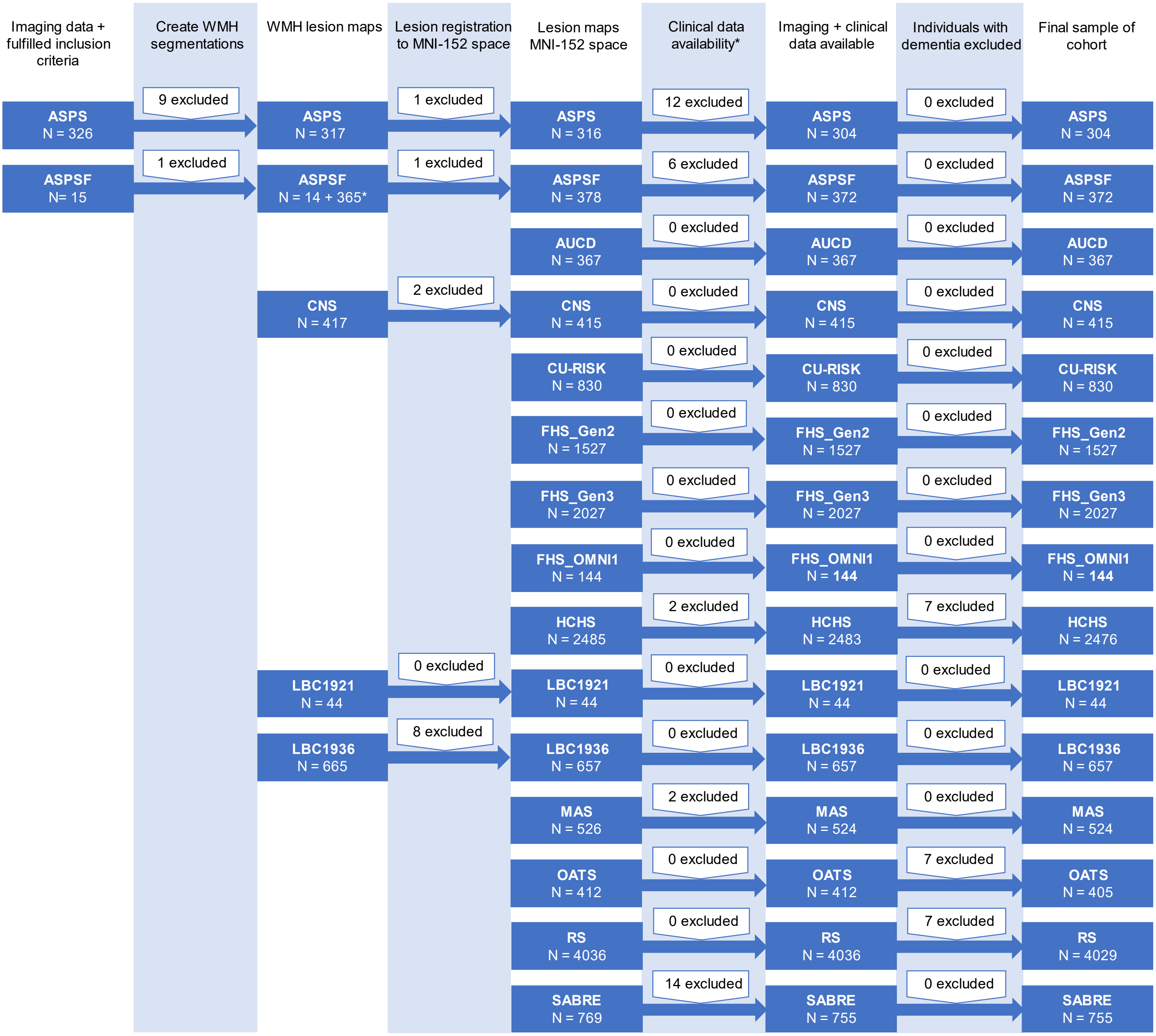

Figure S1. Flowchart of participant selection
Flowchart of participant selection stratified by cohort. *Availability of clinical data on age, sex and diagnosis of dementia. **For ASPSF, 15 WMH segmentations were performed centrally and the other 365 segmentations were performed by the cohort. Abbreviations: ASPS, Austrian Stroke Prevention Study; ASPSF, Austrian Stroke Prevention Family Study; AUCD, UC Davis Alzheimer's Disease Center Diversity Cohort; CNS, Calgary Normative Study; CU-RISK, Chinese University of Hong Kong- Risk Index for Subclinical brain lesions in Hong Kong; FHS, Framingham Heart Study; Gen2, Offspring cohort; Gen3, Third generation cohort; OMNI, minorities cohort; HCHS, Hamburg City Health Study; LBC1921, Lothian Birth Cohort 1921; LBC1936, Lothian Birth Cohort 1936; MAS, Sydney Memory and Ageing Study; OATS, Older Australian Twins Study; RS, Rotterdam Study; SABRE, Southall And Brent Revisited.

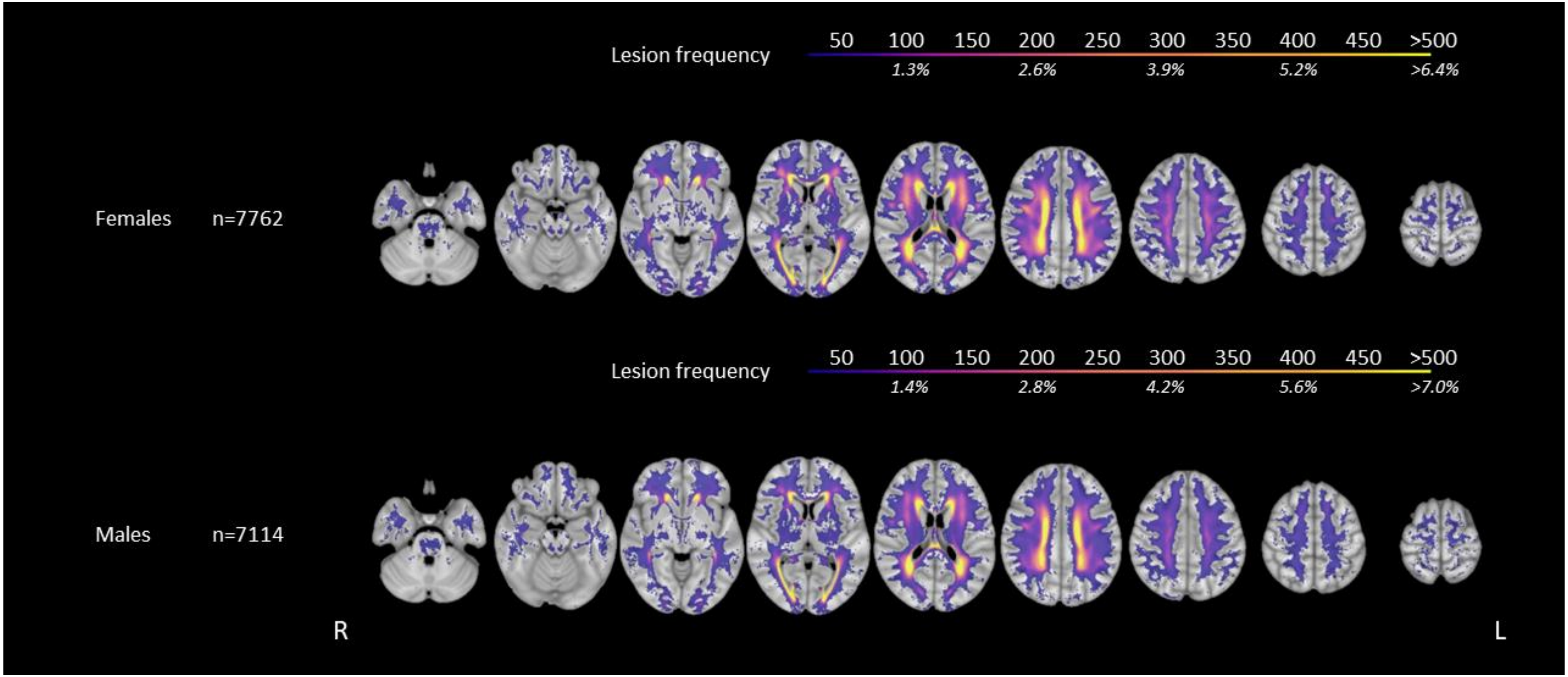

Figure S2. Lesion prevalence maps stratified by sex
Prevalence map of white matter hyperintensities stratified by sex. This figure shows how often each location in the brain was affected by white matter hyperintensities for each of the sexes. Displayed by radiological convention. The plasma color scale from the plasma color palette in R (version 4.1.2) was used for visualization. Abbreviations: L, Left; R, Right.

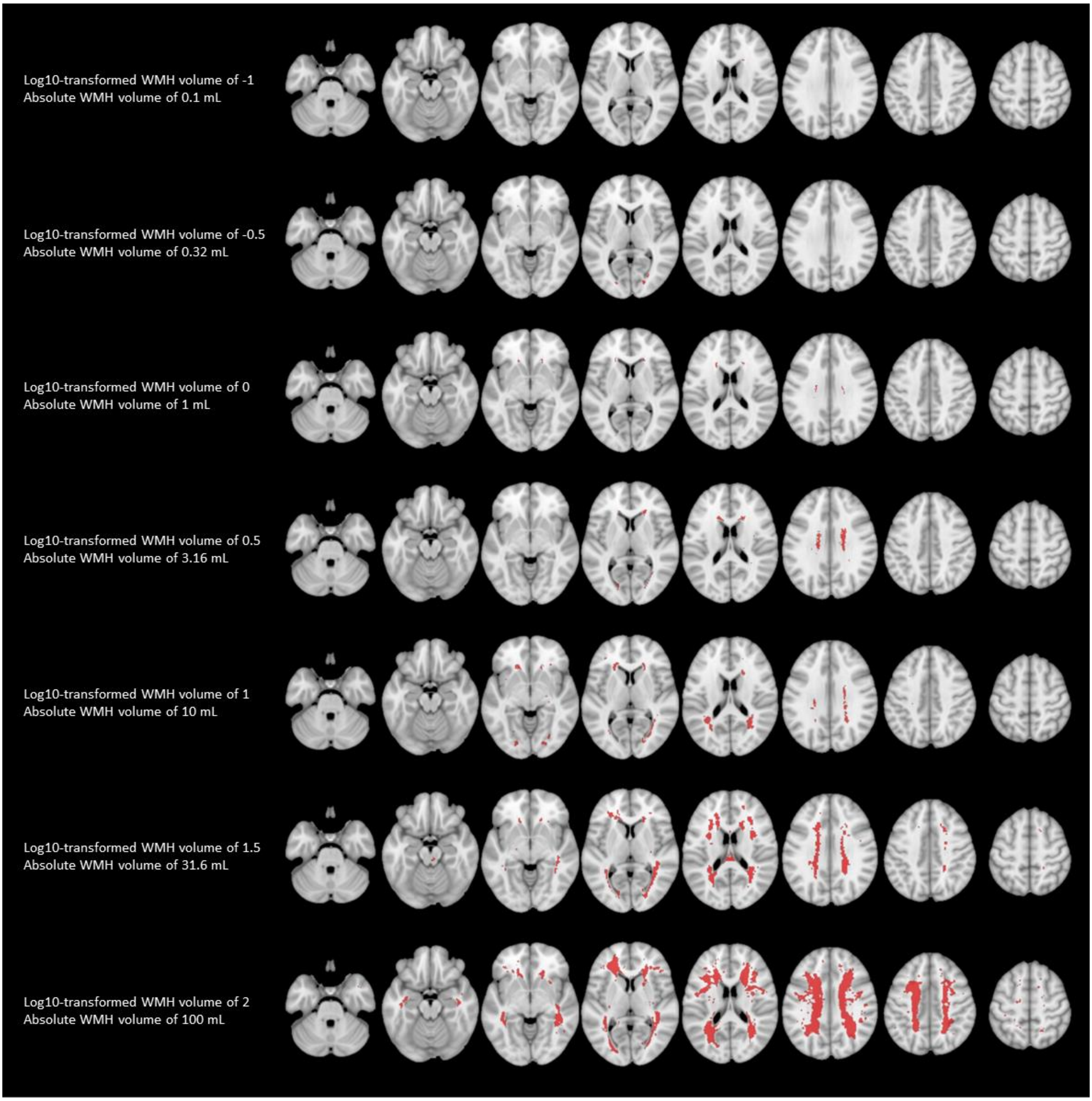

Figure S3. Examples of white matter hyperintensity distributions corresponding to log10-transformed volumes

This figure shows examples of white matter hyperintensity distributions corresponding to the log10-transformed white matter hyperintensity volumes of -1, -0.5, 0, 0.5, 1, 1.5 and 2 respectively. Absolute volumes are provided as a reference. All rows reflect a lesion map of a single participant (n=1) who matched the log10-transformed volume. Abbreviations: WMH, white matter hyperintensities

Table S2: Baseline, stratified by cohort

Variable	Measure	ASPS	ASPSF	AUCD	CNS	CU-RISK	FHS_Gen2	FHS_Gen3	FHS_OMNI1	HCHS	LBC1921	LBC1936	MAS	OATS	RS	SABRE	Overall
n		304	372	367	415	830	1527	2027	144	2476	44	657	524	405	4029	755	14876
Age (y)	mean (SD)	70.5 (6.3)	65.1 (10.7)	74.0 (7.1)	49.3 (17.3)	71.4 (5.1)	66.3 (8.8)	47.9 (8.7)	61.4 (7.9)	63.9 (8.3)	91.7 (0.5)	72.2 (0.8)	77.9 (4.6)	70.2 (5.0)	64.3 (10.5)	70.6 (6.8)	63.9 (11.9)
Sex (female)	n (%)	190 (62.5)	223 (59.9)	247 (67.3)	235 (56.6)	322 (38.8)	815 (53.4)	1081 (53.3)	90 (62.5)	1103 (44.5)	23 (52.3)	307 (46.7)	286 (54.6)	264 (65.2)	2235 (55.5)	341 (45.2)	7762 (52.2)
Race and ethnicity #	missing				†		†	†					†				†
	white, n (%)	304 (100.0)	372 (100.0)	153 (41.7)	338 (81.8)		1501 (99.0)	2002 (99.0)		2476 (100.0)	44 (100.0)	657 (100.0)	511 (97.9)	405 (100.0)	Estimated ~97%	318 (42.1)	13014 (88.3)
	black, n (%)			96 (26.2)	2 (0.5)		2 (0.1)	3 (0.1)	50 (34.7)							178 (23.6)	333 (2.3)
	asian, n (%)			11 (3.0)	63 (15.3)	830 (100.0)	1 (0.1)						8 (1.5)			255 (33.8)	1168 (7.9)
	other, n (%)			107 (29.2)	10 (2.4)		12 (0.8)	17 (0.8)	94 (65.3)				3 (0.6)			4 (0.5)	220 (1.5)
Educational level ##	missing									†					†	*	†
	lower, n (%)	93 (30.6)	68 (18.3)	126 (34.3)	46 (11.1)	710 (85.5)	42 (2.8)	8 (0.4)	12 (8.3)	66 (2.7)	24 (54.5)	364 (55.4)	325 (62.0)	269 (66.4)	325 (8.1)	495 (83.3)	2973 (20.3)
	intermediate, n (%)	121 (39.8)	164 (44.1)	10 (2.7)	195 (47.0)	27 (3.3)	396 (25.9)	258 (12.7)	19 (13.2)	630 (26.1)	16 (36.4)	293 (44.6)	34 (6.5)	21 (5.2)	2761 (69.2)	81 (13.6)	5026 (34.4)
	higher, n (%)	90 (29.6)	140 (37.6)	231 (62.9)	174 (41.9)	93 (11.2)	1089 (71.3)	1761 (86.9)	113 (78.5)	1721 (71.2)	4 (9.1)		165 (31.5)	115 (28.4)	903 (22.6)	18 (3.0)	6617 (45.3)
Hypertension	n (%)	257 (84.5)	270 (72.6)	220 (64.7) ‡	60 (14.5)	645 (77.7)	812 (53.2)	420 (20.8) †	70 (48.6)	1719 (71.8) †	30 (68.2)	325 (49.5)	301 (57.7) †	275 (68.8) †	2477 (61.6) †	547 (72.5) †	8428 (57.2) †
Diabetes mellitus	n (%)	47 (15.5)	39 (10.5)	101 (30.0) ‡	19 (4.6)	197 (23.7)	210 (14.0) †	89 (4.4) †	23 (17.0) ‡	211 (9.1) ‡	1 (2.3)	66 (10.0)	56 (10.7) †	41 (10.1) †	1079 (26.9) †	158 (21.1) †	2337 (16.0) †
Hypercholes-terolemia	n (%)	254 (83.6)	281 (75.5)	176 (55.7) *	58 (14.0)	802 (96.6)	128 (8.4) †	119 (5.9) †	12 (8.4) †	617 (26.4) ‡	15 (34.1)	277 (42.2)	310 (59.4) †	213 (53.2) †	1929 (48.2) †	386 (51.2) †	5577 (38.1) †
Smoking habit	missing		†	-				†		†			†	†	†	*	†
	never, n (%)	206 (67.8)	194 (53.0)		281 (67.7)	677 (81.6)	1404 (91.9)	1846 (91.1)	140 (97.2)	2039 (82.6)	22 (50.0)	311 (47.3)	252 (48.3)	261 (64.9)	1211 (30.2)	405 (62.0)	9249 (64.4)
	current, n (%)	29 (9.5)	55 (15.0)		34 (8.2)	29 (3.5)	123 (8.1)	180 (8.9)	4 (2.8)	429 (17.4)	3 (6.8)	54 (8.2)	20 (3.8)	19 (4.7)	763 (19.0)	22 (3.4)	1764 (12.3)
	past, n (%)	69 (22.7)	117 (32.0)		100 (24.1)	124 (14.9)					19 (43.2)	292 (44.4)	250 (47.9)	122 (30.3)	2032 (50.7)	226 (34.6)	3351 (23.3)
BMI (kg/m^2)	median (IQR)	26.5 (23.8-29.4)	25.7 (23.6-28.7)	-	-	23.3 (21.3-25.4) †	27.5 (24.5-30.8) †	27.0 (24.0-30.7) †	26.6 (24.2-30.4)	26.2 (23.6-29.0) †	-	27.4 (25.0-30.2)	24.8 (22.8-27.6) *	26.1 (23.7-28.6) †	26.9 (24.7-29.6) †	27.2 (24.6-30.4)	26.5 (24.0-29.6) ‡
Atrial fibrillation	n (%)	-	-	14 (5.3) *	-	15 (1.8)	79 (5.2)	22 (1.1)	3 (2.1)	139 (6.1) ‡	-	-	23 (4.4) †	17 (4.3) †	-	19 (3.0) *	331 (3.8) *
Cardio-vascular disease	n (%)	0 (0.0)	0 (0.0)	52 (18.2) *	-	49 (5.9)	176 (11.5)	39 (1.9)	17 (11.8)	84 (3.4) †	21 (47.7)	182 (27.7)	201 (38.6) †	109 (27.2) †	-	74 (10.0) †	1004 (9.7) *
History of stroke	n (%)	0 (0.0)	0 (0.0)	23 (6.6) †	0 (0.0)	0 (0.0)	0 (0.0)	0 (0.0)	0 (0.0)	81 (3.3) †	4 (9.3) †	45 (6.8)	12 (2.3) †	14 (3.5) †	28 (0.7)	26 (3.5) †	233 (1.6) †
WMH volume (ml)	median (IQR)	4.0 (1.7-8.5)	5.7 (3.0-11.6)	3.7 (1.3-11.3)	0.9 (0.5-2.0)	2.8 (1.3-6.6)	1.6 (0.7-3.0)	0.4 (0.2-0.7)	0.7 (0.3-1.7)	1.7 (0.8-3.8)	47.6 (30.1-75.9)	10.1 (4.6-21.9)	9.3 (4.8-16.9)	3.0 (1.4-5.7)	2.7 (1.6-5.1)	1.1 (0.3-1.9)	2.0 (0.8-4.8)

Missing data: † <5%; ‡ 5-10%; *10-50%;** >50%, # the group other race and ethnicity includes different categories for each cohort, details are described at p.6 of the supplemental material. ## lower: less than high school completion, intermediate: high school completion, higher: all education beyond high school completion. Abbreviations: BMI, Body Mass Index; IQR, interquartile range; kg, kilograms; m, meters; mL, milliliter; SD, standard deviation; WMH, white matter hyperintensities.

5-year age strata and inter- and intracohort observations

Numbers of participants for each sex-specific 5-year age-stratum in the total population

Age strata	Males, n	Females, n
40-44	168	216
45-49	478	487
50-54	630	780
55-59	780	907
60-64	858	1005
**65-69**	**1281**	**1247**
**70-74**	**1464**	**1471**
75-79	790	802
80-84	312	397

A. Comparisons between individual cohorts and the total population: comparisons of median WMH volumes of the two largest cohorts (HCHS and RS) with the merged dataset for the two most represented 5-year age-strata (65-69 and 70-74 years).

	HCHS	RS
Males	Median WMH volumes on average 15% lower	Median WMH volumes on average 25% higher
Females	Median WMH volumes ranging from 15% lower to 10% higher	Median WMH volumes on average 23% higher

B. Comparisons within individual cohorts: comparisons of the 25th and 75th percentiles WMH volumes with the 50th percentile (median) within the individual cohorts (HCHS and RS) and within the merged dataset for the two most represented 5-year age-strata (64-69 and 70-74 years). Percentages reflect the increase or decrease in WMH volume in relation to the median WMH volumes.

Total population (merged dataset)

Males	25th percentile	50th percentile	75th percentile
65-69 (n=1281)	1,04 (↓47%)	1,97	3,85 (↑95%)
70-74 (n=1464)	1,60 (↓56%)	3,63	8,00 (↑120%)
Females			
65-69 (n=1247)	1,18 (↓50%)	2,34	4,40 (↑88%)
70-74 (n=1471)	2,18 (↓52%)	4,55	10,62 (↑133%)

HCHS

Males	25th percentile	50th percentile	75th percentile
65-69 (n=148)	0,91 (↓50%)	1,82	3,82 (↑110%)
70-74 (n=102)	1,47 (↓48%)	2,84	5,09 (↑79%)
Females			
65-69 (n=213)	1,27 (↓51%)	2,58	5,26 (↑104%)
70-74 (n=251)	2,13 (↓45%)	3,87	7,36 (↑90%)

RS

Males	25th percentile	50th percentile	75th percentile
65-69 (n=310)	1,77 (↓33%)	2,66	4,33 (↑63%)
70-74 (n=314)	2,56 (↓39%)	4,17	6,45 (↑55%)
Females			
65-69 (n=270)	2,14 (↓33%)	3,18	5,11 (↑61%)
70-74 (n=283)	3,04 (↓39%)	5,0	8,20 (↑64%)

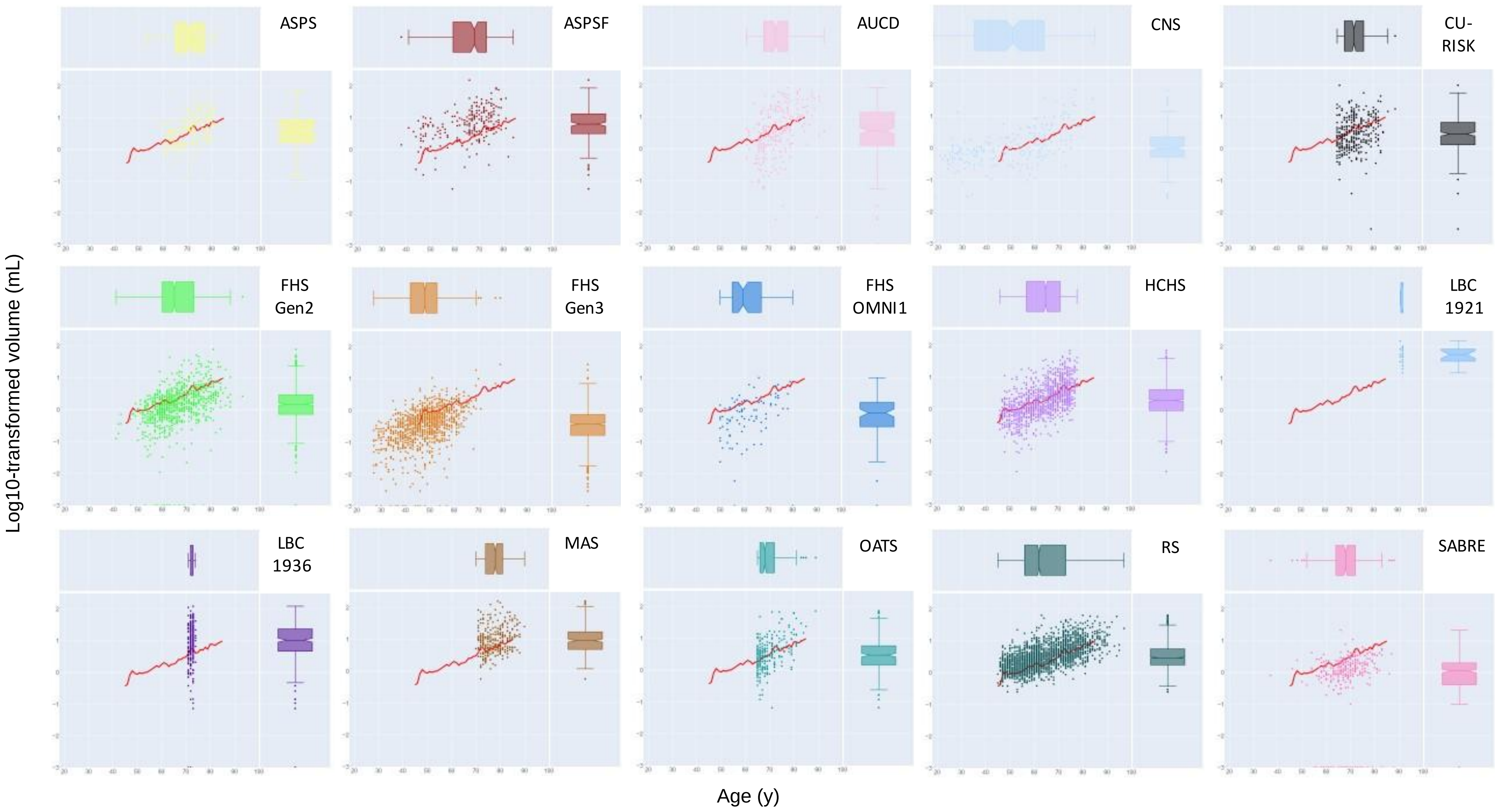

Figure S4-1. Cohort-specific distribution of log10-transformed white matter hyperintensity volume versus age, in females
This figure shows datapoints for log10-transformed white matter hyperintensity volumes versus age for females, stratified by cohort. The red line reflects median values of the merged cohort for each age, as a reference. Color-mapping was based on the 15-color palette for color blindness by Martin Krzywinski, accessed via https://mk.bcgsc.ca/biovis2012/ on June 20th 2023. Abbreviations: mL, milliliter; y, years

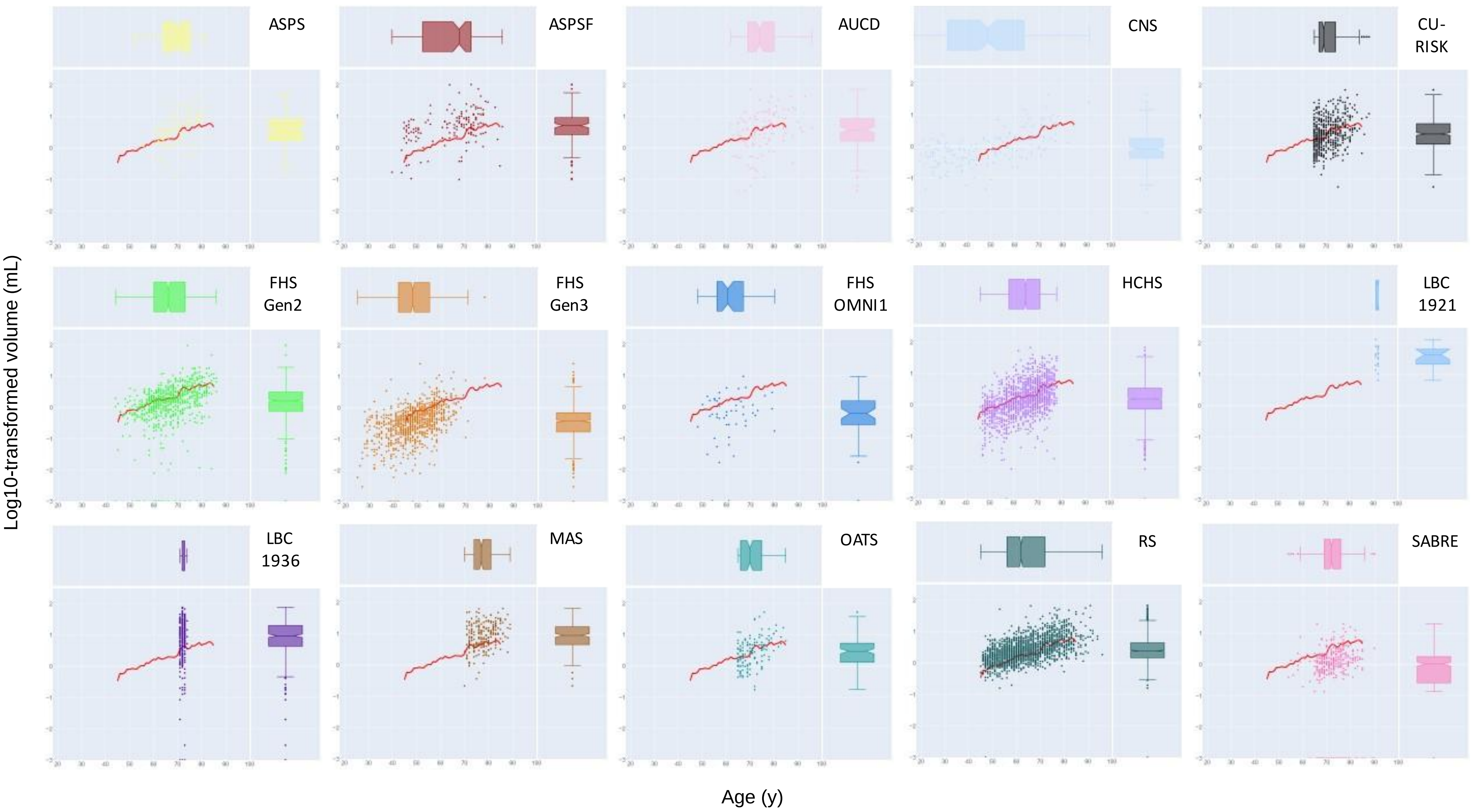

Figure S4-2. Cohort-specific distribution of log10-transformed white matter hyperintensity volume versus age, in males
This figure shows datapoints for log10-transformed white matter hyperintensity volumes versus age for males, stratified by cohort. The red line reflects median values of the merged cohort for each age, as a reference. Color-mapping was based on the 15-color palette for color blindness by Martin Krzywinski, accessed via https://mk.bcgsc.ca/biovis2012/ on June 20th 2023. Abbreviations: mL, milliliter; y, years

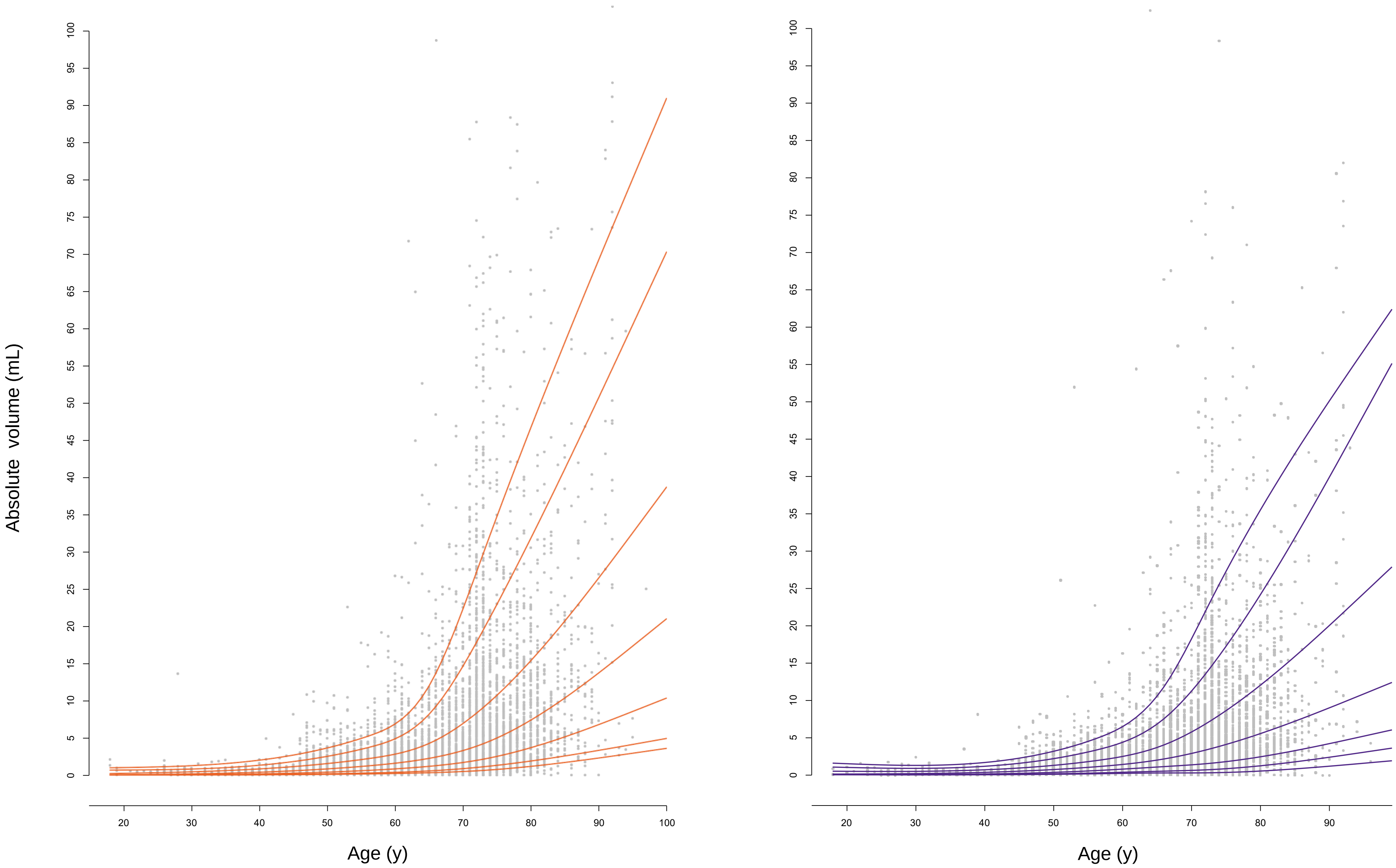

Figure S5. Centile curves for total absolute white matter hyperintensity volume, stratified by sex.
5th, 10th, 25th, 50th, 75th, 90th, and 95th centile curves for females (left, in orange) and males (right, in purple) respectively. Color-mapping was based on contrasting colors for color blindness by David Nichols, accessed via https://davidmathlogic.com/colorblind on June 20th 2023. Abbreviations: mL, milliliter; y, years

Sensitivity analyses

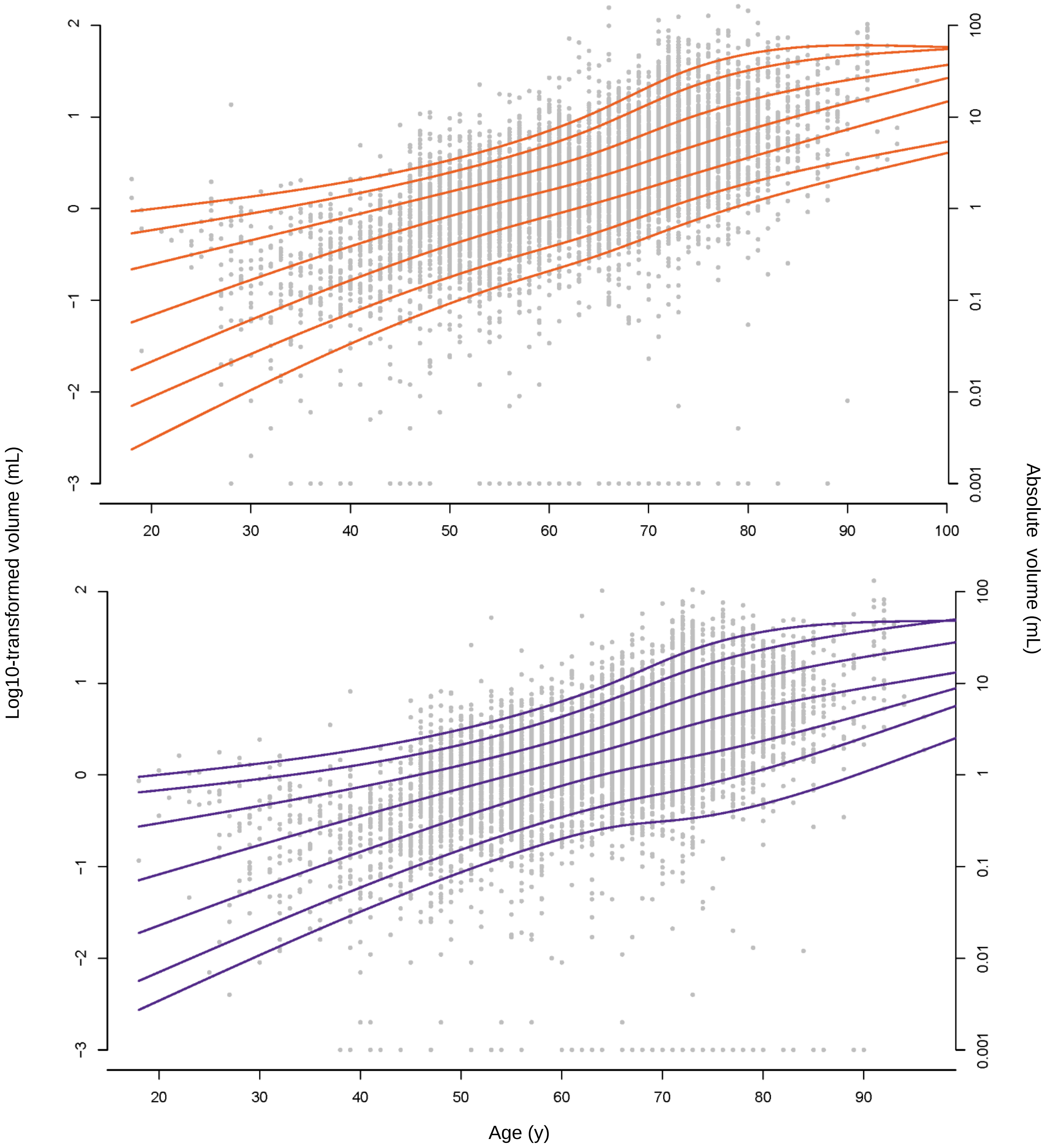

Figure S6. Centile curves for WMH volumes, stratified by sex, for the stroke-free population
5th, 10th, 25th, 50th, 75th, 90th, and 95th centile curves for females (upper figure, in orange) and males (lower figure, in purple) respectively, after exclusion of individuals with a history of stroke. Color-mapping was based on contrasting colors for color blindness by David Nichols, accessed via https://davidmathlogic.com/colorblind on June 20th 2023. Abbreviations: mL, milliliter; y, years

Table S3: Normative values for absolute total white matter hyperintensity volume, by age and sex after exclusion of individuals with a history of stroke

	Percentiles						
	p5	p10	p25	p50	p75	p90	p95
**Age (years)**	**Females**						
40	0.03	0.07	0.16	0.39	0.83	1.41	1.99
45	0.06	0.11	0.26	0.57	1.12	1.84	2.54
50	0.09	0.18	0.40	0.82	1.53	2.47	3.38
55	0.14	0.26	0.59	1.16	2.08	3.44	4.74
60	0.21	0.38	0.83	1.58	2.86	5.00	7.09
65	0.31	0.56	1.17	2.22	4.19	7.93	11.70
70	0.49	0.86	1.70	3.31	6.72	13.78	21.24
75	0.77	1.32	2.48	4.98	10.59	22.68	35.46
80	1.14	1.89	3.58	7.24	15.17	32.16	48.81
85	1.62	2.56	5.13	10.24	20.11	40.37	57.46
**Age (years)**	**Males**						
40	0.03	0.06	0.14	0.35	0.74	1.29	1.91
45	0.05	0.10	0.22	0.50	0.96	1.63	2.39
50	0.09	0.15	0.34	0.71	1.28	2.13	3.14
55	0.13	0.23	0.52	1.00	1.74	2.95	4.33
60	0.20	0.35	0.76	1.39	2.45	4.32	6.34
65	0.27	0.48	1.06	1.96	3.62	6.71	10.05
70	0.31	0.63	1.37	2.85	5.62	10.90	17.25
75	0.36	0.82	1.76	4.04	8.44	16.78	27.26
80	0.48	1.15	2.36	5.45	11.74	23.38	36.50
85	0.69	1.68	3.25	7.05	15.38	30.11	42.98

Percentiles were derived from quantile regression models for the specific ages shown. Volumes are absolute normalized white matter hyperintensity volumes in MNI-152 space, shown in milliliter. Abbreviations: p, percentile.

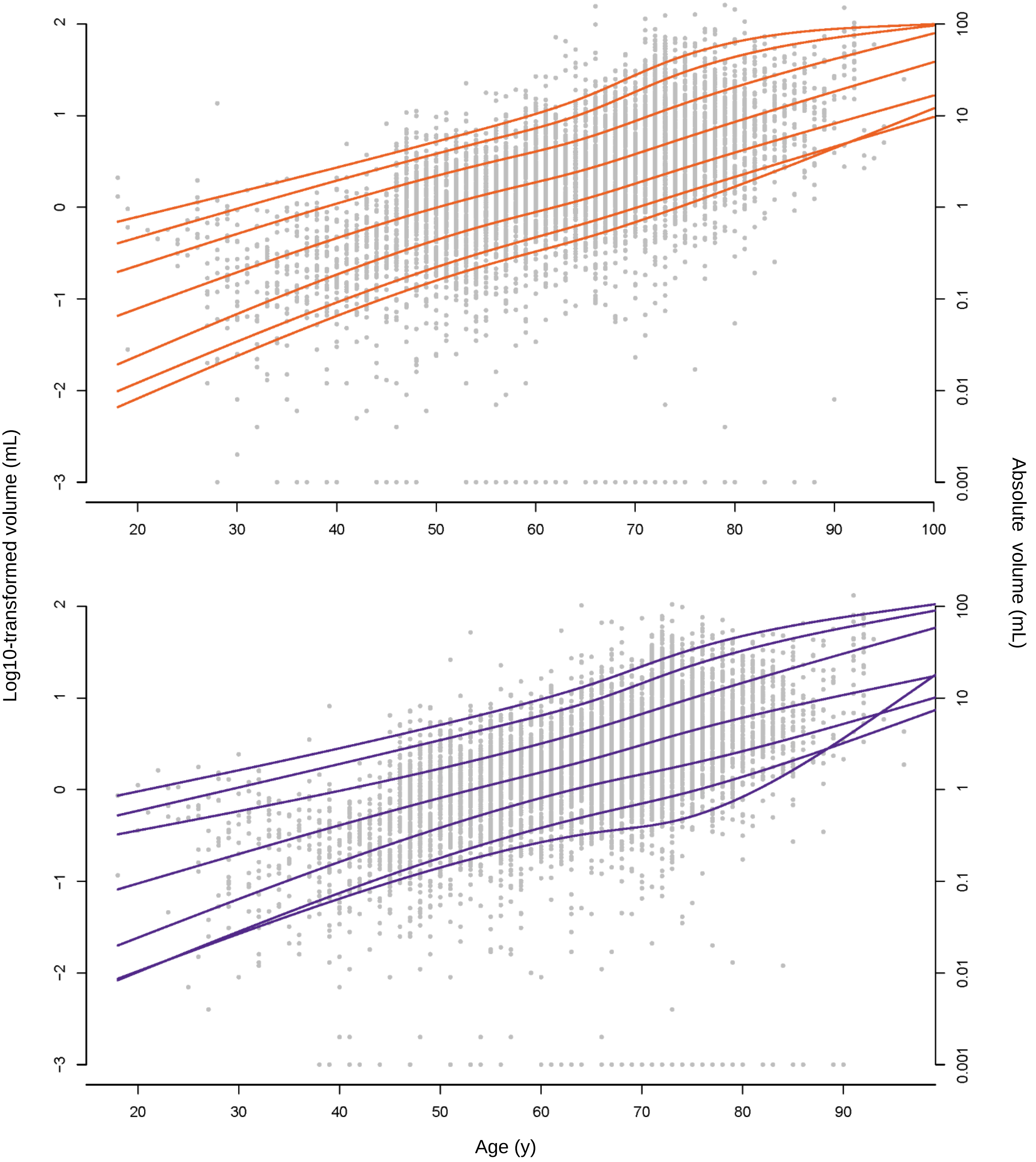

Figure S7. Centile curves for WMH volumes, stratified by sex, with correction for image processing pipeline
5th, 10th, 25th, 50th, 75th, 90th, and 95th centile curves for females (upper figure, in orange) and males (lower figure, in purple) respectively. The model included image processing pipeline as covariate in the quantile regression, with RegLSM use as reference category. Color-mapping was based on contrasting colors for color blindness by David Nichols, accessed via https://davidmathlogic.com/colorblind on June 20th 2023. Abbreviations: mL, milliliter; y, years

Table S4: normative values for absolute total white matter hyperintensity volume, by age and sex, with correction for image processing pipeline.

	Percentiles						
	**p5**	**p10**	**p25**	**p50**	**p75**	**p90**	**p95**
**Age (years)**	**Females**						
40	0.06	0.09	0.18	0.46	1.10	1.95	2.71
45	0.10	0.14	0.29	0.68	1.57	2.75	3.74
50	0.16	0.22	0.44	0.99	2.20	3.84	5.20
55	0.23	0.33	0.64	1.38	3.02	5.29	7.30
60	0.33	0.47	0.89	1.87	4.05	7.28	10.43
65	0.46	0.67	1.24	2.58	5.68	10.78	16.08
70	0.69	0.98	1.81	3.80	8.73	18.16	27.67
75	1.06	1.45	2.69	5.73	13.65	30.00	45.30
80	1.67	2.14	3.95	8.53	20.43	43.77	63.46
85	2.70	3.14	5.71	12.55	29.47	57.66	78.28
**Age (years)**	**Males**						
40	0.06	0.07	0.16	0.41	0.97	1.90	2.82
45	0.10	0.12	0.25	0.58	1.27	2.57	3.77
50	0.14	0.18	0.38	0.81	1.70	3.47	5.08
55	0.20	0.27	0.57	1.13	2.30	4.70	6.95
60	0.27	0.38	0.81	1.54	3.18	6.43	9.68
65	0.33	0.53	1.12	2.14	4.53	9.23	14.12
70	0.39	0.71	1.47	3.08	6.70	14.47	22.10
75	0.51	0.96	1.93	4.41	9.99	22.65	33.91
80	0.83	1.38	2.63	6.09	14.66	32.84	47.46
85	1.64	2.08	3.67	8.19	21.23	44.70	61.65

Percentiles were derived from quantile regression models for the specific ages shown. The model included image processing pipeline as covariate in the quantile regression, with RegLSM use as reference category. Volumes are absolute normalized white matter hyperintensity volumes in MNI-152 space, shown in milliliter. Abbreviations: p, percentile.

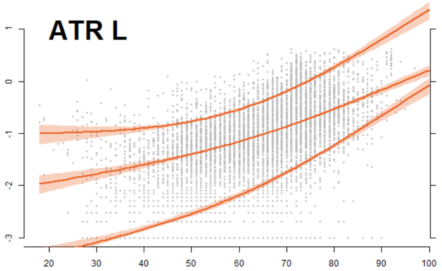

age	p10	p50	p90
40	0.00	0.02	0.12
45	0.00	0.03	0.14
50	0.00	0.04	0.17
55	0.00	0.05	0.21
60	0.01	0.07	0.29
65	0.01	0.09	0.41
70	0.02	0.13	0.64
75	0.03	0.20	1.06
80	0.06	0.29	1.85
85	0.11	0.44	3.38

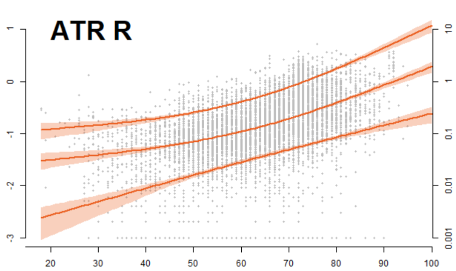

age	p10	p50	p90
40	0.01	0.05	0.18
45	0.01	0.06	0.21
50	0.01	0.07	0.25
55	0.02	0.08	0.31
60	0.03	0.11	0.41
65	0.04	0.14	0.55
70	0.05	0.19	0.78
75	0.06	0.27	1.15
80	0.08	0.39	1.76
85	0.11	0.57	2.77

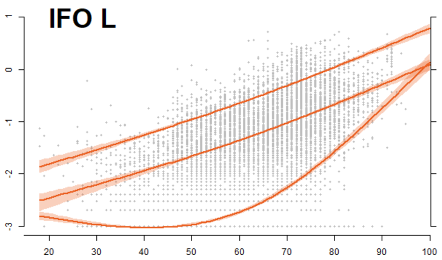

age	p10	p50	p90
40	0.00	0.01	0.05
45	0.00	0.01	0.08
50	0.00	0.02	0.11
55	0.00	0.03	0.15
60	0.00	0.04	0.22
65	0.00	0.06	0.33
70	0.00	0.09	0.48
75	0.01	0.14	0.73
80	0.03	0.21	1.10
85	0.07	0.33	1.68

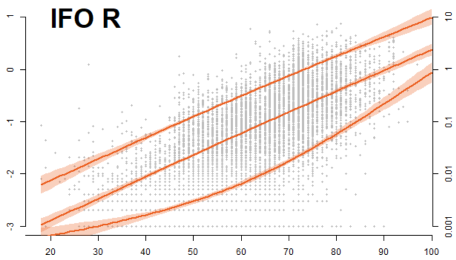

age	p10	p50	p90
40	0.00	0.01	0.05
45	0.00	0.01	0.08
50	0.00	0.02	0.12
55	0.00	0.04	0.20
60	0.01	0.06	0.31
65	0.01	0.09	0.48
70	0.02	0.15	0.75
75	0.03	0.24	1.16
80	0.06	0.38	1.79
85	0.11	0.60	2.74

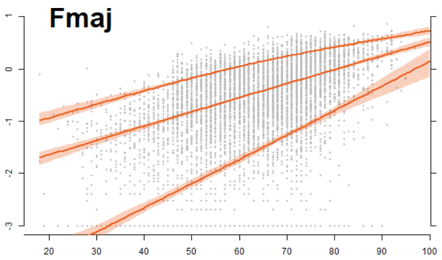

age	p10	p50	p90
40	0.00	0.08	0.38
45	0.00	0.11	0.50
50	0.01	0.15	0.66
55	0.01	0.21	0.87
60	0.02	0.28	1.11
65	0.03	0.38	1.41
70	0.05	0.52	1.75
75	0.09	0.71	2.16
80	0.16	0.97	2.62
85	0.27	1.31	3.15

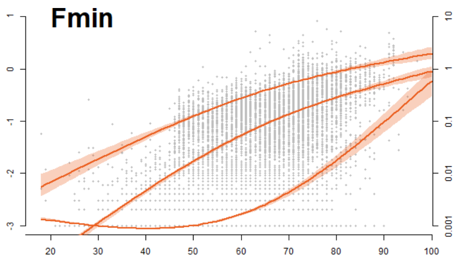

age	p10	p50	p90
40	0.00	0.00	0.05
45	0.00	0.01	0.08
50	0.00	0.02	0.12
55	0.00	0.03	0.19
60	0.00	0.05	0.27
65	0.00	0.09	0.39
70	0.00	0.13	0.53
75	0.01	0.20	0.69
80	0.02	0.28	0.88
85	0.04	0.39	1.09

Figure S8-1: Centile curves and absolute white matter hyperintensity volumes for curve pattern 1 in females
The figures show the 10th, 50th and 90th centile curves of log10-transformed white matter hyperintensity volumes with 95% CI and corresponding absolute volumes in milliliter for white matter tracts (based on the JHU atlas) that follow curve pattern 1 (tracts following the same curve as total white matter hyperintensity volume) in females. Abbreviations: ATR, anterior thalamic tract; Fmaj, forceps major; Fmin, forceps minor; IFO, inferior fronto-occipital fasciculus; L, left; R, right

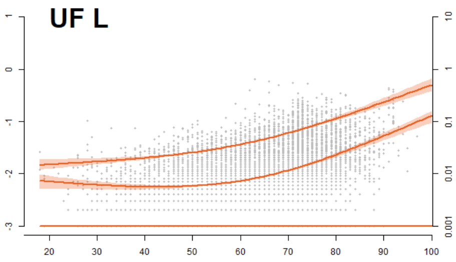

age	p10	p50	p90
40	0.00	0.00	0.02
45	0.00	0.00	0.02
50	0.00	0.00	0.02
55	0.00	0.01	0.03
60	0.00	0.01	0.04
65	0.00	0.01	0.04
70	0.00	0.01	0.06
75	0.00	0.01	0.08
80	0.00	0.02	0.11
85	0.00	0.03	0.16

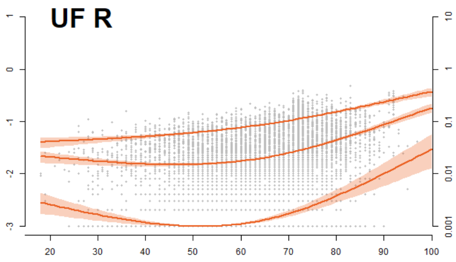

age	p10	p50	p90
40	0.00	0.01	0.05
45	0.00	0.01	0.05
50	0.00	0.01	0.06
55	0.00	0.01	0.07
60	0.00	0.02	0.08
65	0.00	0.02	0.09
70	0.00	0.02	0.10
75	0.00	0.03	0.12
80	0.00	0.04	0.15
85	0.01	0.06	0.19

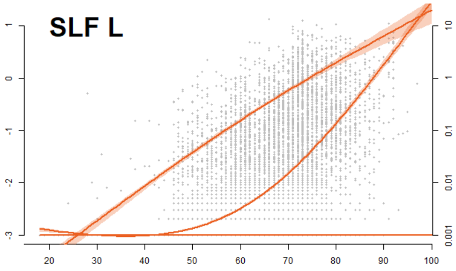

age	p10	p50	p90
40	0.00	0.00	0.01
45	0.00	0.00	0.02
50	0.00	0.00	0.04
55	0.00	0.00	0.08
60	0.00	0.00	0.15
65	0.00	0.01	0.30
70	0.00	0.01	0.58
75	0.00	0.04	1.08
80	0.00	0.13	1.98
85	0.00	0.48	3.57

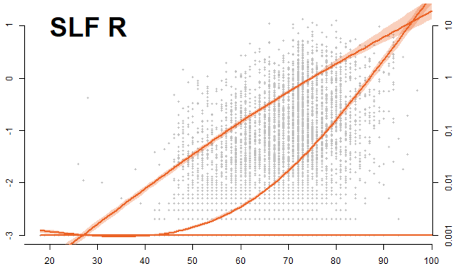

age	p10	p50	p90
40	0.00	0.00	0.01
45	0.00	0.00	0.02
50	0.00	0.00	0.03
55	0.00	0.00	0.07
60	0.00	0.00	0.14
65	0.00	0.01	0.29
70	0.00	0.02	0.55
75	0.00	0.05	1.03
80	0.00	0.16	1.89
85	0.00	0.58	3.41

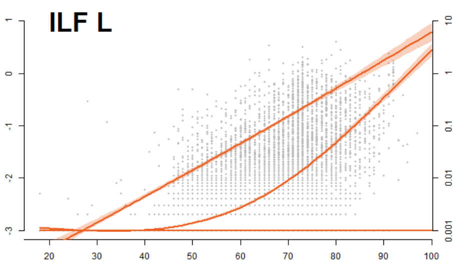

age	p10	p50	p90
40	0.00	0.00	0.00
45	0.00	0.00	0.01
50	0.00	0.00	0.01
55	0.00	0.00	0.02
60	0.00	0.00	0.04
65	0.00	0.00	0.08
70	0.00	0.01	0.15
75	0.00	0.02	0.28
80	0.00	0.05	0.51
85	0.00	0.12	0.95

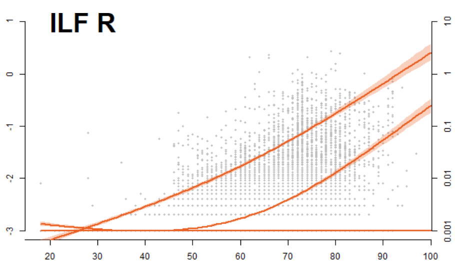

age	p10	p50	p90
40	0.00	0.00	0.00
45	0.00	0.00	0.00
50	0.00	0.00	0.01
55	0.00	0.00	0.01
60	0.00	0.00	0.02
65	0.00	0.00	0.03
70	0.00	0.00	0.05
75	0.00	0.01	0.09
80	0.00	0.01	0.17
85	0.00	0.02	0.33

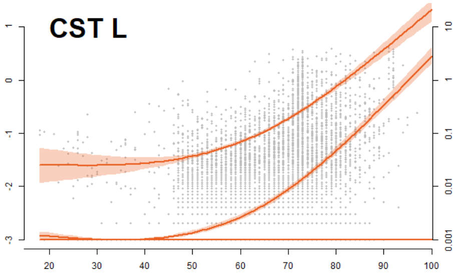

age	p10	p50	p90
40	0.00	0.00	0.03
45	0.00	0.00	0.03
50	0.00	0.00	0.04
55	0.00	0.00	0.05
60	0.00	0.00	0.07
65	0.00	0.00	0.11
70	0.00	0.01	0.19
75	0.00	0.02	0.36
80	0.00	0.05	0.75
85	0.00	0.12	1.64

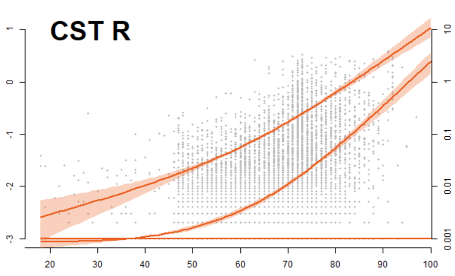

age	p10	p50	p90
40	0.00	0.00	0.01
45	0.00	0.00	0.01
50	0.00	0.00	0.02
55	0.00	0.00	0.03
60	0.00	0.00	0.06
65	0.00	0.00	0.09
70	0.00	0.01	0.17
75	0.00	0.02	0.32
80	0.00	0.05	0.62
85	0.00	0.13	1.24

Figure S8-2: Centile curves and absolute white matter hyperintensity volumes for curve pattern 2 in females
The figures show the 10th, 50th and 90th centile curves of log10-transformed white matter hyperintensity volumes with 95% CI and corresponding absolute volumes in milliliter for white matter tracts (based on the JHU atlas) that follow curve pattern 2 (tracts where white matter hyperintensity accumulation started around the age of 60-65 years and accelerated at higher ages) in females. Abbreviations: CST, corticospinal tract; ILF, inferior longitudinal fasciculus; SLF,

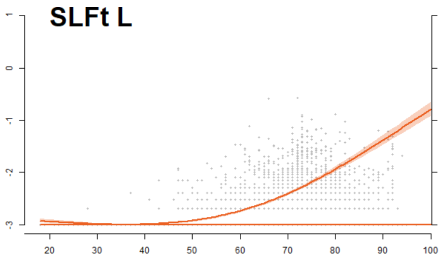

age	p10	p50	p90
40	0.00	0.00	0.00
45	0.00	0.00	0.00
50	0.00	0.00	0.00
55	0.00	0.00	0.00
60	0.00	0.00	0.00
65	0.00	0.00	0.00
70	0.00	0.00	0.00
75	0.00	0.00	0.01
80	0.00	0.00	0.01
85	0.00	0.00	0.02

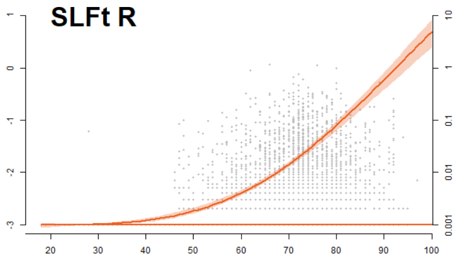

age	p10	p50	p90
40	0.00	0.00	0.00
45	0.00	0.00	0.00
50	0.00	0.00	0.00
55	0.00	0.00	0.00
60	0.00	0.00	0.00
65	0.00	0.00	0.01
70	0.00	0.00	0.01
75	0.00	0.00	0.03
80	0.00	0.00	0.08
85	0.00	0.00	0.21

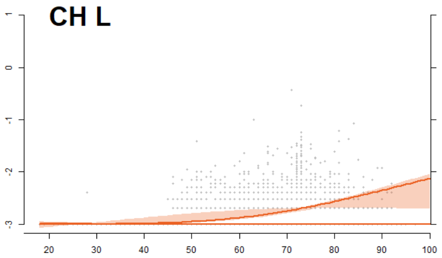

age	p10	p50	p90
40	0.00	0.00	0.00
45	0.00	0.00	0.00
50	0.00	0.00	0.00
55	0.00	0.00	0.00
60	0.00	0.00	0.00
65	0.00	0.00	0.00
70	0.00	0.00	0.00
75	0.00	0.00	0.00
80	0.00	0.00	0.00
85	0.00	0.00	0.00

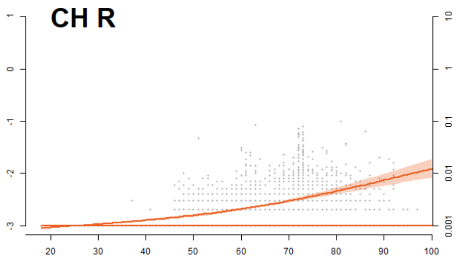

age	p10	p50	p90
40	0.00	0.00	0.00
45	0.00	0.00	0.00
50	0.00	0.00	0.00
55	0.00	0.00	0.00
60	0.00	0.00	0.00
65	0.00	0.00	0.00
70	0.00	0.00	0.00
75	0.00	0.00	0.00
80	0.00	0.00	0.00
85	0.00	0.00	0.00

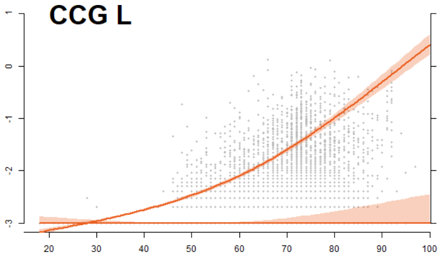

age	p10	p50	p90
40	0.00	0.00	0.00
45	0.00	0.00	0.00
50	0.00	0.00	0.00
55	0.00	0.00	0.00
60	0.00	0.00	0.01
65	0.00	0.00	0.01
70	0.00	0.00	0.02
75	0.00	0.00	0.05
80	0.00	0.00	0.10
85	0.00	0.00	0.22

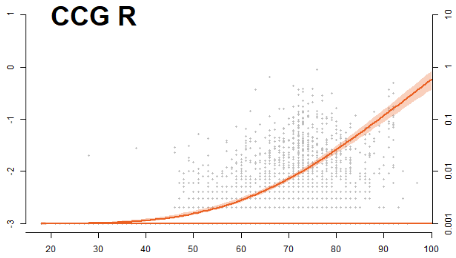

age	p10	p50	p90
40	0.00	0.00	0.00
45	0.00	0.00	0.00
50	0.00	0.00	0.00
55	0.00	0.00	0.00
60	0.00	0.00	0.00
65	0.00	0.00	0.00
70	0.00	0.00	0.01
75	0.00	0.00	0.01
80	0.00	0.00	0.02
85	0.00	0.00	0.05

Figure S8-3: Centile curves and absolute white matter hyperintensity volumes for curve pattern 3 in females
The figures show the 10th, 50th and 90th centile curves of log10-transformed white matter hyperintensity volumes with 95% CI and corresponding absolute volumes in milliliter for white matter tracts (based on the JHU atlas) that follow curve pattern 3 (tracts where white matter hyperintensity were rare even at higher ages) in females. Abbreviations: CCG, cingulum cingulate gyrus; CH, cingulum- hippocampal part; SLF temp, superior longitudinal fasciculus- temporal part; L, left; R, right

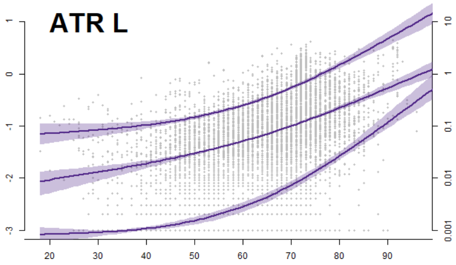

age	p10	p50	p90
40	0.00	0.02	0.10
45	0.00	0.02	0.12
50	0.00	0.03	0.15
55	0.00	0.04	0.19
60	0.00	0.05	0.25
65	0.00	0.07	0.36
70	0.01	0.10	0.54
75	0.01	0.15	0.88
80	0.03	0.22	1.49
85	0.05	0.34	2.62

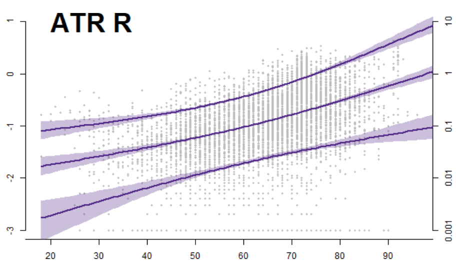

age	p10	p50	p90
40	0.01	0.04	0.15
45	0.01	0.05	0.18
50	0.01	0.06	0.22
55	0.01	0.07	0.28
60	0.02	0.09	0.36
65	0.02	0.12	0.49
70	0.03	0.16	0.69
75	0.04	0.22	1.01
80	0.05	0.30	1.52
85	0.06	0.42	2.34

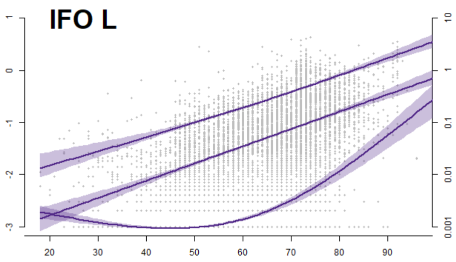

age	p10	p50	p90
40	0.00	0.01	0.05
45	0.00	0.01	0.07
50	0.00	0.02	0.10
55	0.00	0.02	0.14
60	0.00	0.03	0.19
65	0.00	0.05	0.27
70	0.00	0.07	0.38
75	0.00	0.11	0.55
80	0.01	0.16	0.80
85	0.02	0.23	1.17

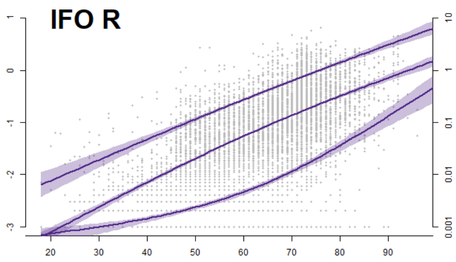

age	p10	p50	p90
40	0.00	0.01	0.05
45	0.00	0.01	0.07
50	0.00	0.02	0.11
55	0.00	0.03	0.18
60	0.00	0.05	0.27
65	0.01	0.09	0.42
70	0.01	0.14	0.63
75	0.02	0.21	0.95
80	0.04	0.32	1.41
85	0.07	0.48	2.09

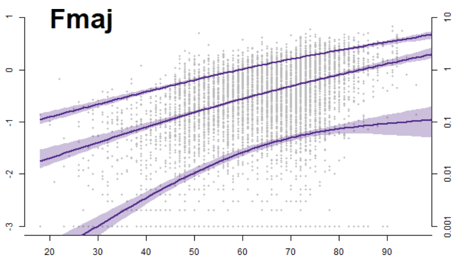

age	p10	p50	p90
40	0.00	0.08	0.37
45	0.01	0.11	0.49
50	0.01	0.15	0.63
55	0.02	0.21	0.81
60	0.02	0.28	1.03
65	0.04	0.37	1.29
70	0.05	0.49	1.60
75	0.06	0.63	1.96
80	0.07	0.81	2.37
85	0.08	1.03	2.85

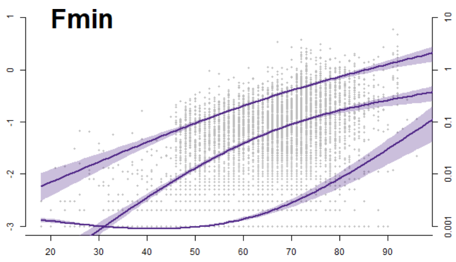

age	p10	p50	p90
40	0.00	0.00	0.04
45	0.00	0.01	0.06
50	0.00	0.01	0.09
55	0.00	0.02	0.14
60	0.00	0.04	0.20
65	0.00	0.06	0.29
70	0.00	0.09	0.40
75	0.00	0.12	0.55
80	0.01	0.16	0.73
85	0.01	0.21	0.97

Figure S8-4: Centile curves and absolute white matter hyperintensity volumes for curve pattern 1 in males
The figures show the 10th, 50th and 90th centile curves of log10-transformed white matter hyperintensity volumes with 95% CI and corresponding absolute volumes in milliliter for white matter tracts (based on the JHU atlas) that follow curve pattern 1 (tracts following the same curve as total white matter hyperintensity volume) in males. Abbreviations: ATR, anterior thalamic tract; Fmaj, forceps major; Fmin, forceps minor; IFO, inferior fronto-occipital fasciculus; L, left; R, right

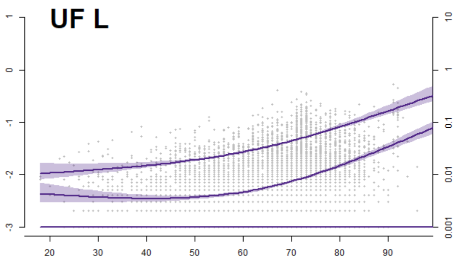

age	p10	p50	p90
40	0.00	0.00	0.01
45	0.00	0.00	0.02
50	0.00	0.00	0.02
55	0.00	0.00	0.02
60	0.00	0.00	0.03
65	0.00	0.00	0.03
70	0.00	0.01	0.04
75	0.00	0.01	0.06
80	0.00	0.01	0.08
85	0.00	0.02	0.11

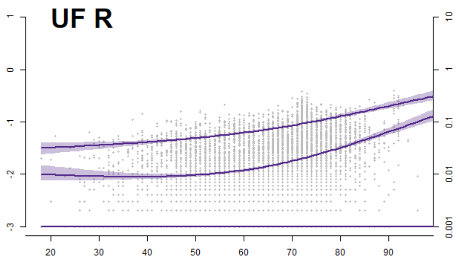

age	p10	p50	p90
40	0.00	0.01	0.04
45	0.00	0.01	0.04
50	0.00	0.01	0.05
55	0.00	0.01	0.05
60	0.00	0.01	0.06
65	0.00	0.01	0.07
70	0.00	0.02	0.08
75	0.00	0.02	0.10
80	0.00	0.03	0.13
85	0.00	0.04	0.16

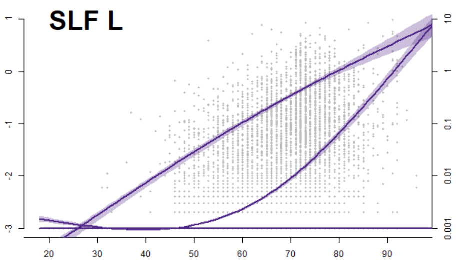

age	p10	p50	p90
40	0.00	0.00	0.01
45	0.00	0.00	0.01
50	0.00	0.00	0.03
55	0.00	0.00	0.05
60	0.00	0.00	0.10
65	0.00	0.00	0.19
70	0.00	0.01	0.35
75	0.00	0.02	0.61
80	0.00	0.07	1.06
85	0.00	0.21	1.80

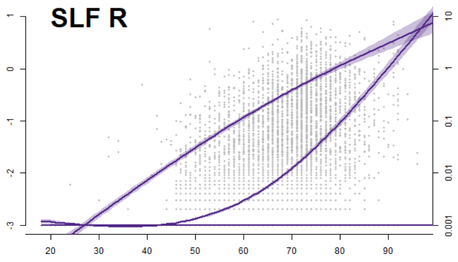

age		p50	p90
40		0.00	0.01
45		0.00	0.01
50		0.00	0.03
55		0.00	0.06
60		0.00	0.12
65		0.00	0.22
70		0.01	0.39
75		0.03	0.68
80		0.09	1.15
85		0.31	1.91

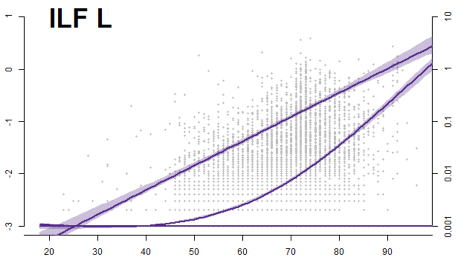

age	p10	p50	p90
40	0.00	0.00	0.00
45	0.00	0.00	0.01
50	0.00	0.00	0.01
55	0.00	0.00	0.02
60	0.00	0.00	0.04
65	0.00	0.00	0.07
70	0.00	0.01	0.12
75	0.00	0.01	0.21
80	0.00	0.03	0.35
85	0.00	0.09	0.60

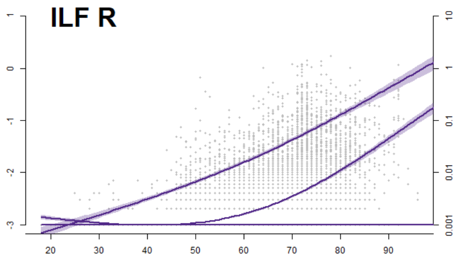

age	p10	p50	p90
40	0.00	0.00	0.00
45	0.00	0.00	0.00
50	0.00	0.00	0.01
55	0.00	0.00	0.01
60	0.00	0.00	0.01
65	0.00	0.00	0.02
70	0.00	0.00	0.04
75	0.00	0.01	0.07
80	0.00	0.01	0.13
85	0.00	0.02	0.23

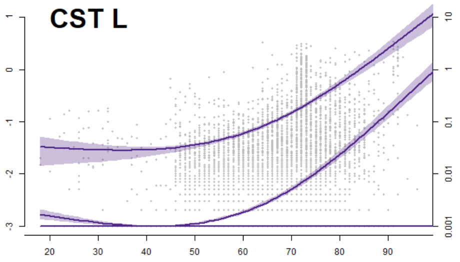

age	p10	p50	p90
40	0.00	0.00	0.03
45	0.00	0.00	0.03
50	0.00	0.00	0.03
55	0.00	0.00	0.04
60	0.00	0.00	0.06
65	0.00	0.00	0.09
70	0.00	0.00	0.15
75	0.00	0.01	0.27
80	0.00	0.02	0.54
85	0.00	0.06	1.15

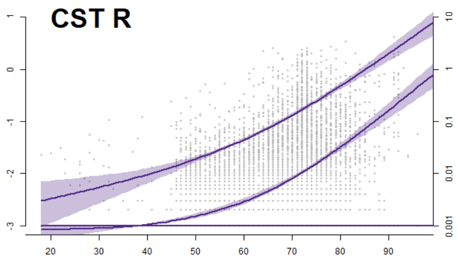

age	p10	p50	p90
40	0.00	0.00	0.01
45	0.00	0.00	0.01
50	0.00	0.00	0.02
55	0.00	0.00	0.03
60	0.00	0.00	0.04
65	0.00	0.00	0.07
70	0.00	0.01	0.13
75	0.00	0.01	0.24
80	0.00	0.03	0.48
85	0.00	0.07	0.97

Figure S8-5: Centile curves and absolute white matter hyperintensity volumes for curve pattern 2 in males
The figures show the 10th, 50th and 90th centile curves of log10-transformed white matter hyperintensity volumes with 95% CI and corresponding absolute volumes in milliliter for white matter tracts (based on the JHU atlas) that follow curve pattern 2 (tracts where white matter hyperintensity accumulation started around the age of 60-65 years and accelerated at higher ages) in males. Abbreviations: CST, corticospinal tract; ILF, inferior longitudinal fasciculus; SLF, superior

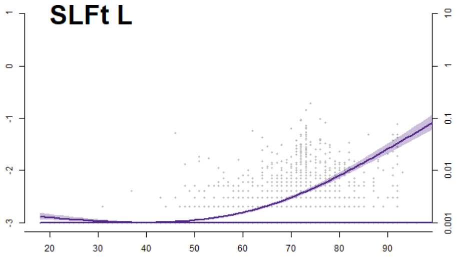

age	p10	p50	p90
40	0.00	0.00	0.00
45	0.00	0.00	0.00
50	0.00	0.00	0.00
55	0.00	0.00	0.00
60	0.00	0.00	0.00
65	0.00	0.00	0.00
70	0.00	0.00	0.00
75	0.00	0.00	0.00
80	0.00	0.00	0.01
85	0.00	0.00	0.01

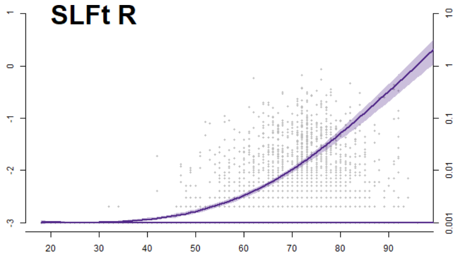

age	p10	p50	p90
40	0.00	0.00	0.00
45	0.00	0.00	0.00
50	0.00	0.00	0.00
55	0.00	0.00	0.00
60	0.00	0.00	0.00
65	0.00	0.00	0.00
70	0.00	0.00	0.01
75	0.00	0.00	0.02
80	0.00	0.00	0.05
85	0.00	0.00	0.13

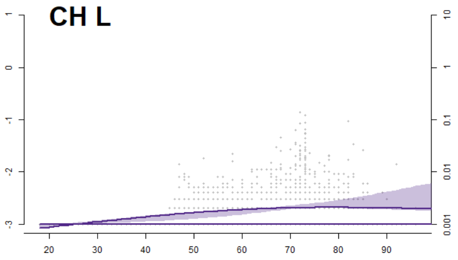

age	p10	p50	p90
40	0.00	0.00	0.00
45	0.00	0.00	0.00
50	0.00	0.00	0.00
55	0.00	0.00	0.00
60	0.00	0.00	0.00
65	0.00	0.00	0.00
70	0.00	0.00	0.00
75	0.00	0.00	0.00
80	0.00	0.00	0.00
85	0.00	0.00	0.00

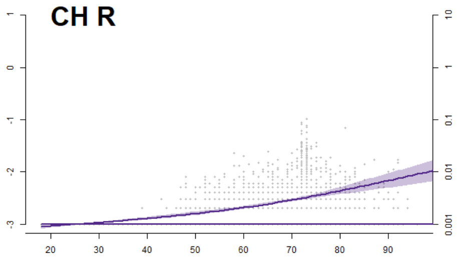

age	p10	p50	p90
40	0.00	0.00	0.00
45	0.00	0.00	0.00
50	0.00	0.00	0.00
55	0.00	0.00	0.00
60	0.00	0.00	0.00
65	0.00	0.00	0.00
70	0.00	0.00	0.00
75	0.00	0.00	0.00
80	0.00	0.00	0.00
85	0.00	0.00	0.00

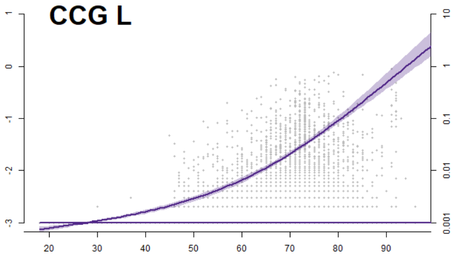

age	p10	p50	p90
40	0.00	0.00	0.00
45	0.00	0.00	0.00
50	0.00	0.00	0.00
55	0.00	0.00	0.00
60	0.00	0.00	0.01
65	0.00	0.00	0.01
70	0.00	0.00	0.02
75	0.00	0.00	0.04
80	0.00	0.00	0.09
85	0.00	0.00	0.21

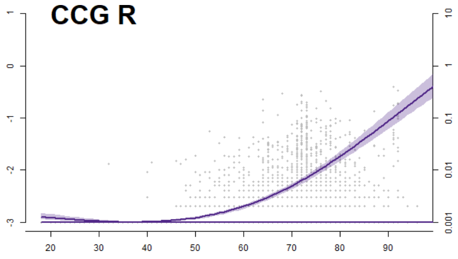

age	p10	p50	p90
40	0.00	0.00	0.00
45	0.00	0.00	0.00
50	0.00	0.00	0.00
55	0.00	0.00	0.00
60	0.00	0.00	0.00
65	0.00	0.00	0.00
70	0.00	0.00	0.00
75	0.00	0.00	0.01
80	0.00	0.00	0.02
85	0.00	0.00	0.04

Figure S8-6: Centile curves and absolute white matter hyperintensity volumes for curve pattern 3 in males
The figures show the 10th, 50th and 90th centile curves of log10-transformed white matter hyperintensity volumes with 95% CI and corresponding absolute volumes in milliliter for white matter tracts (based on the JHU atlas) that follow curve pattern 3 (tracts where white matter hyperintensity were rare even at higher ages) in males. Abbreviations: CCG, cingulum cingulate gyrus; CH, cingulum- hippocampal part; SLF temp, superior longitudinal fasciculus- temporal part; L, left; R, right

References

Biesbroek, J.M., Kuijf, H.J., Weaver, N.A., Zhao, L., Duering, M., Biessels, G.J., 2019. Brain Infarct Segmentation and Registration on MRI or CT for Lesion-symptom Mapping. Journal of Visualized Experiments 2019. https://doi.org/10.3791/59653

Biesbroek, J.M., Lam, B.Y.K., Zhao, L., Tang, Y., Wang, Z., Abrigo, J., Chu, W.W.C., Wong, A., Shi, L., Kuijf, H.J., Biessels, G.J., Mok, V.C.T., 2020. High white matter hyperintensity burden in strategic white matter tracts relates to worse global cognitive performance in community-dwelling individuals. J Neurol Sci 414. https://doi.org/10.1016/j.jns.2020.116835

Camarasa R, Doué C, de Bruijne M, Dubost F., 2018. Segmentation of White Matter Hyperintensities with an Ensemble of Multi-Dimensional Convolutional Gated Recurrent Units. [online] Available: https://wmh.isi.uu.nl/wp-content/uploads/2018/08/coroflo.pdf. [WWW Document]. URL https://wmh.isi.uu.nl/wp-content/uploads/2018/08/coroflo.pdf (accessed 5.31.21).

de Boer, R., Vrooman, H.A., van der Lijn, F., Vernooij, M.W., Ikram, M.A., van der Lugt, A., Breteler, M.M.B., Niessen, W.J., 2009. White matter lesion extension to automatic brain tissue segmentation on MRI. Neuroimage 45, 1151–1161. https://doi.org/10.1016/j.neuroimage.2009.01.011

Deary, I.J., Gow, A.J., Taylor, M.D., Corley, J., Brett, C., Wilson, V., Campbell, H., Whalley, L.J., Visscher, P.M., Porteous, D.J., Starr, J.M., 2007. The Lothian Birth Cohort 1936: a study to examine influences on cognitive ageing from age 11 to age 70 and beyond. BMC Geriatr 7, 28. https://doi.org/10.1186/1471-2318-7-28

Deary, I.J., Whiteman, M.C., Starr, J.M., Whalley, L.J., Fox, H.C., 2004. The Impact of Childhood Intelligence on Later Life: Following Up the Scottish Mental Surveys of 1932 and 1947. J Pers Soc Psychol 86, 130–147. https://doi.org/10.1037/0022-3514.86.1.130

DeCarli, C., Fletcher, E., Ramey, V., Harvey, D., Jagust, W.J., 2005. Anatomical Mapping of White Matter Hyperintensities (WMH). Stroke 36, 50–55. https://doi.org/10.1161/01.STR.0000150668.58689.f2

DeCarli, C., Miller, B.L., Swan, G.E., Reed, T., Wolf, P.A., Garner, J., Jack, L., Carmelli, D., 1999. Predictors of Brain Morphology for the Men of the NHLBI Twin Study. Stroke 30, 529–536. https://doi.org/10.1161/01.STR.30.3.529

Feinleib, M., Kannel, W.B., Garrison, R.J., McNamara, P.M., Castelli, W.P., 1975. The framingham offspring study. Design and preliminary data. Prev Med (Baltim) 4, 518–525. https://doi.org/10.1016/0091-7435(75)90037-7

Fletcher, E., 2014. Using Prior Information To Enhance Sensitivity of Longitudinal Brain Change Computation, in: Frontiers of Medical Imaging. WORLD SCIENTIFIC, pp. 63–81. https://doi.org/10.1142/9789814611107_0004

Fletcher, E., DeCarli, C., Fan, A.P., Knaack, A., 2021. Convolutional Neural Net Learning Can Achieve Production-Level Brain Segmentation in Structural Magnetic Resonance Imaging. Front Neurosci 15. https://doi.org/10.3389/fnins.2021.683426

Fletcher, E., Singh, B., Harvey, D., Carmichael, O., DeCarli, C., 2012. Adaptive image segmentation for robust measurement of longitudinal brain tissue change, in: 2012

Annual International Conference of the IEEE Engineering in Medicine and Biology Society. IEEE, pp. 5319–5322. https://doi.org/10.1109/EMBC.2012.6347195

Fonov, V., Evans, A.C., Botteron, K., Almli, C.R., McKinstry, R.C., Collins, D.L., 2011. Unbiased average age-appropriate atlases for pediatric studies. Neuroimage 54, 313–327. https://doi.org/10.1016/j.neuroimage.2010.07.033

Gobbi D, Lu Q, Frayne R, Salluzzi M, 2012. Cerebra-WML: a rapid workflow for quantification of white matter hyperintensities. Canadian Stroke Congress 40, E128–E129.

Good, C.D., Johnsrude, I.S., Ashburner, J., Henson, R.N.A., Friston, K.J., Frackowiak, R.S.J., 2001. A Voxel-Based Morphometric Study of Ageing in 465 Normal Adult Human Brains. Neuroimage 14, 21–36. https://doi.org/10.1006/nimg.2001.0786

Griffanti, L., Zamboni, G., Khan, A., Li, L., Bonifacio, G., Sundaresan, V., Schulz, U.G., Kuker, W., Battaglini, M., Rothwell, P.M., Jenkinson, M., 2016. BIANCA (Brain Intensity AbNormality Classification Algorithm): A new tool for automated segmentation of white matter hyperintensities. Neuroimage 141, 191–205. https://doi.org/10.1016/j.neuroimage.2016.07.018

Hinton, L., Carter, K., Reed, B.R., Beckett, L., Lara, E., DeCarli, C., Mungas, D., 2010. Recruitment of a Community-based Cohort for Research on Diversity and Risk of Dementia. Alzheimer Dis Assoc Disord 24, 234–241. https://doi.org/10.1097/WAD.0b013e3181c1ee01

Hofman, A., Brusselle, G.G.O., Murad, S.D., van Duijn, C.M., Franco, O.H., Goedegebure, A., Ikram, M.A., Klaver, C.C.W., Nijsten, T.E.C., Peeters, R.P., Stricker, B.H.Ch., Tiemeier, H.W., Uitterlinden, A.G., Vernooij, M.W., 2015. The Rotterdam Study: 2016 objectives and design update. Eur J Epidemiol 30, 661–708. https://doi.org/10.1007/s10654-015-0082-x

Ikram, M.A., van der Lugt, A., Niessen, W.J., Koudstaal, P.J., Krestin, G.P., Hofman, A., Bos, D., Vernooij, M.W., 2015. The Rotterdam Scan Study: design update 2016 and main findings. Eur J Epidemiol 30, 1299–1315. https://doi.org/10.1007/s10654-015-0105-7

Jagodzinski, A., Johansen, C., Koch-Gromus, U., Aarabi, G., Adam, G., Anders, S., Augustin, M., der Kellen, R.B., Beikler, T., Behrendt, C.A., Betz, C.S., Bokemeyer, C., Borof, K., Briken, P., Busch, C.J., Büchel, C., Brassen, S., Debus, E.S., Eggers, L., Fiehler, J., Gallinat, J., Gellißen, S., Gerloff, C., Girdauskas, E., Gosau, M., Graefen, M., Härter, M., Harth, V., Heidemann, C., Heydecke, G., Huber, T.B., Hussein, Y., Kampf, M.O., von dem Knesebeck, O., Konnopka, A., König, H.H., Kromer, R., Kubisch, C., Kühn, S., Loges, S., Löwe, B., Lund, G., Meyer, C., Nagel, L., Nienhaus, A., Pantel, K., Petersen, E., Püschel, K., Reichenspurner, H., Sauter, G., Scherer, M., Scherschel, K., Schiffner, U., Schnabel, R.B., Schulz, H., Smeets, R., Sokalskis, V., Spitzer, M.S., Terschüren, C., Thederan, I., Thoma, T., Thomalla, G., Waschki, B., Wegscheider, K., Wenzel, J.P., Wiese, S., Zyriax, B.C., Zeller, T., Blankenberg, S., 2020. Rationale and Design of the Hamburg City Health Study. Eur J Epidemiol 35, 169–181. https://doi.org/10.1007/s10654-019-00577-4

Kochunov, P., Lancaster, J.L., Thompson, P., Woods, R., Mazziotta, J., Hardies, J., Fox, P., 2001. Regional Spatial Normalization: Toward an Optimal Target. J Comput Assist Tomogr 25, 805–816. https://doi.org/10.1097/00004728-200109000-00023

Kuijf, H.J., Biesbroek, J.M., De Bresser, J., Heinen, R., Andermatt, S., Bento, M., Berseth, M., Belyaev, M., Cardoso, M.J., Casamitjana, A., Collins, D.L., Dadar, M., Georgiou, A., Ghafoorian, M., Jin, D., Khademi, A., Knight, J., Li, H., Lladó, X., Luna, M., Mahmood, Q., McKinley, R., Mehrtash, A., Ourselin, S., Park, B.-Y., Park, H., Park, S.H., Pezold, S., Puybareau, E., Rittner, L., Sudre, C.H., Valverde, S., Vilaplana, V., Wiest, R., Xu, Y., Xu, Z., Zeng, G., Zhang, J., Zheng, G., Chen, C., van der Flier, W., Barkhof, F., Viergever, M.A., Biessels, G.J., 2019. Standardized Assessment of Automatic Segmentation of White Matter Hyperintensities and Results of the WMH Segmentation Challenge. IEEE Trans Med Imaging 38, 2556–2568. https://doi.org/10.1109/TMI.2019.2905770

Lo, J.W., Crawford, J.D., Desmond, D.W., Godefroy, O., Jokinen, H., Mahinrad, S., Bae, H.J., Lim, J.S., Köhler, S., Douven, E., Staals, J., Chen, C., Xu, X., Chong, E.J., Akinyemi, R.O., Kalaria, R.N., Ogunniyi, A., Barbay, M., Roussel, M., Lee, B.C., Srikanth, V.K., Moran, C., Kandiah, N., Chander, R.J., Sabayan, B., Jukema, J.W., Melkas, S., Erkinjuntti, T., Brodaty, H., Bordet, R., Bombois, S., Hénon, H., Lipnicki, D.M., Kochan, N.A., Sachdev, P.S., 2019. Profile of and risk factors for poststroke cognitive impairment in diverse ethnoregional groups. Neurology 93, E2257–E2271. https://doi.org/10.1212/WNL.0000000000008612

Maillard, P., Lu, H., Arfanakis, K., Gold, B.T., Bauer, C.E., Zachariou, V., Stables, L., Wang, D.J.J., Jann, K., Seshadri, S., Duering, M., Hillmer, L.J., Rosenberg, G.A., Snoussi, H., Sepehrband, F., Habes, M., Singh, B., Kramer, J.H., Corriveau, R.A., Singh, H., Schwab, K., Helmer, K.G., Greenberg, S.M., Caprihan, A., DeCarli, C., Satizabal, C.L., 2022. Instrumental validation of free water, peak-width of skeletonized mean diffusivity, and white matter hyperintensities: MarkVCID neuroimaging kits. Alzheimer’s & Dementia: Diagnosis, Assessment & Disease Monitoring 14. https://doi.org/10.1002/dad2.12261

McCreary, C.R., Salluzzi, M., Andersen, L.B., Gobbi, D., Lauzon, L., Saad, F., Smith, E.E., Frayne, R., 2020. Calgary Normative Study: design of a prospective longitudinal study to characterise potential quantitative MR biomarkers of neurodegeneration over the adult lifespan. BMJ Open 10, e038120. https://doi.org/10.1136/bmjopen-2020-038120

Modat, M., Cash, D.M., Daga, P., Winston, G.P., Duncan, J.S., Ourselin, S., 2014. Global image registration using a symmetric block-matching approach. Journal of Medical Imaging 1, 024003. https://doi.org/10.1117/1.JMI.1.2.024003

Modat, M., Ridgway, G.R., Taylor, Z.A., Lehmann, M., Barnes, J., Hawkes, D.J., Fox, N.C., Ourselin, S., 2010. Fast free-form deformation using graphics processing units. Comput Methods Programs Biomed 98, 278–284. https://doi.org/10.1016/j.cmpb.2009.09.002

Petersen, M., Frey, B.M., Mayer, C., Kühn, S., Gallinat, J., Hanning, U., Fiehler, J., Borof, K., Jagodzinski, A., Gerloff, C., Thomalla, G., Cheng, B., 2022. Fixel based analysis of white matter alterations in early stage cerebral small vessel disease. Sci Rep 12, 1581. https://doi.org/10.1038/s41598-022-05665-2

Petersen, M., Frey, B.M., Schlemm, E., Mayer, C., Hanning, U., Engelke, K., Fiehler, J., Borof, K., Jagodzinski, A., Gerloff, C., Thomalla, G., Cheng, B., 2020. Network Localisation of White Matter Damage in Cerebral Small Vessel Disease. Sci Rep 10. https://doi.org/10.1038/s41598-020-66013-w

Petrea, R.E., Pinheiro, A., Demissie, S., Ekenze, O., Aparicio, H.J., Satizabal, C.L., Maillard, P., DeCarli, C., Beiser, A.S., Seshadri, S., Lioutas, V.-A., Rafael Romero, J., 2024. Hypertension Trends and White Matter Brain Injury in the Offspring Framingham Heart Study Cohort. Hypertension 81, 87–95. https://doi.org/10.1161/HYPERTENSIONAHA.123.21264

Quan, S.F., Howard, B. V, Iber, C., Kiley, J.P., Nieto, F.J., O'Connor, G.T., Rapoport, D.M., Redline, S., Robbins, J., Samet, J.M., Wahl, P.W., 1997. The Sleep Heart Health Study: design, rationale, and methods. Sleep 20, 1077–85.

Ritchie, S.J., Dickie, D.A., Cox, S.R., Valdés Hernández, M. del C., Sibbett, R., Pattie, A., Anblagan, D., Redmond, P., Royle, N.A., Corley, J., Maniega, S.M., Taylor, A.M., Karama, S., Booth, T., Gow, A.J., Starr, J.M., Bastin, M.E., Wardlaw, J.M., Deary, I.J., 2018. Brain structural differences between 73- and 92-year olds matched for childhood intelligence, social background, and intracranial volume. Neurobiol Aging 62, 146–158. https://doi.org/10.1016/j.neurobiolaging.2017.10.005

Rueckert, D., Aljabar, P., Heckemann, R.A., Hajnal, J. V., Hammers, A., 2006. Diffeomorphic Registration Using B-Splines. pp. 702–709. https://doi.org/10.1007/11866763_86

Sachdev, P.S., Brodaty, H., Reppermund, S., Kochan, N.A., Trollor, J.N., Draper, B., Slavin, M.J., Crawford, J., Kang, K., Broe, G.A., Mather, K.A., Lux, O., 2010. The Sydney Memory and Ageing Study (MAS): methodology and baseline medical and neuropsychiatric characteristics of an elderly epidemiological non-demented cohort of Australians aged 70–90 years. Int Psychogeriatr 22, 1248–1264. https://doi.org/10.1017/S1041610210001067

Sachdev, P.S., Lammel, A., Trollor, J.N., Lee, T., Wright, M.J., Ames, D., Wen, W., Martin, N.G., Brodaty, H., Schofield, P.R., 2009. A Comprehensive Neuropsychiatric Study of Elderly Twins: The Older Australian Twins Study. Twin Research and Human Genetics 12, 573–582. https://doi.org/10.1375/twin.12.6.573

Schmidt, R., Fazekas, F., Kapeller, P., Schmidt, H., Hartung, H.-P., 1999. MRI white matter hyperintensities. Neurology 53, 132–132. https://doi.org/10.1212/WNL.53.1.132

Schmidt, R., Lechner, H., Fazekas, F., Niederkorn, K., Reinhart, B., Grieshofer, P., Horner, S., Offenbacher, H., Koch, M., Eber, B., Schumacher, M., Kapeller, P., Freidl, W., Dusek, T., 1994. Assessment of Cerebrovascular Risk Profiles in Healthy Persons: Definition of Research Goals and the Austrian Stroke Prevention Study (ASPS). Neuroepidemiology 13, 308–313. https://doi.org/10.1159/000110396

Seiler, S., Pirpamer, L., Hofer, E., Duering, M., Jouvent, E., Fazekas, F., Mangin, J.F., Chabriat, H., Dichgans, M., Ropele, S., Schmidt, R., 2014. Magnetization transfer ratio relates to cognitive impairment in normal elderly. Front Aging Neurosci 6. https://doi.org/10.3389/fnagi.2014.00263

Shi, L., Wang, D., Liu, S., Pu, Y., Wang, Yilong, Chu, W.C.W., Ahuja, A.T., Wang, Yongjun, 2013. Automated quantification of white matter lesion in magnetic resonance imaging of patients with acute infarction. J Neurosci Methods 213, 138–146. https://doi.org/10.1016/j.jneumeth.2012.12.014

Smith, S.M., Jenkinson, M., Woolrich, M.W., Beckmann, C.F., Behrens, T.E.J., Johansen-Berg, H., Bannister, P.R., De Luca, M., Drobnjak, I., Flitney, D.E., Niazy, R.K.,

Saunders, J., Vickers, J., Zhang, Y., De Stefano, N., Brady, J.M., Matthews, P.M., 2004. Advances in functional and structural MR image analysis and implementation as FSL. Neuroimage 23, S208–S219. https://doi.org/10.1016/j.neuroimage.2004.07.051

Splansky, G.L., Corey, D., Yang, Q., Atwood, L.D., Cupples, L.A., Benjamin, E.J., D’Agostino, R.B., Fox, C.S., Larson, M.G., Murabito, J.M., O’Donnell, C.J., Vasan, R.S., Wolf, P.A., Levy, D., 2007. The Third Generation Cohort of the National Heart, Lung, and Blood Institute’s Framingham Heart Study: Design, Recruitment, and Initial Examination. Am J Epidemiol 165, 1328–1335. https://doi.org/10.1093/aje/kwm021

Sudre, C.H., Cardoso, M.J., Bouvy, W.H., Biessels, G.J., Barnes, J., Ourselin, S., 2015. Bayesian Model Selection for Pathological Neuroimaging Data Applied to White Matter Lesion Segmentation. IEEE Trans Med Imaging 34, 2079–2102. https://doi.org/10.1109/TMI.2015.2419072

Sudre, C.H., Smith, L., Atkinson, D., Chaturvedi, N., Ourselin, S., Barkhof, F., Hughes, A.D., Jäger, H.R., Cardoso, M.J., 2018. Cardiovascular Risk Factors and White Matter Hyperintensities: Difference in Susceptibility in South Asians Compared With Europeans. J Am Heart Assoc 7. https://doi.org/10.1161/JAHA.118.010533

Tillin, T., Forouhi, N.G., McKeigue, P.M., Chaturvedi, N., 2012. Southall And Brent REvisited: Cohort profile of SABRE, a UK population-based comparison of cardiovascular disease and diabetes in people of European, Indian Asian and African Caribbean origins. Int J Epidemiol 41, 33–42. https://doi.org/10.1093/ije/dyq175

Valdés Hernández, M. del C., Ferguson, K.J., Chappell, F.M., Wardlaw, J.M., 2010. New multispectral MRI data fusion technique for white matter lesion segmentation: method and comparison with thresholding in FLAIR images. Eur Radiol 20, 1684–1691. https://doi.org/10.1007/s00330-010-1718-6

Vrooman, H.A., Cocosco, C.A., van der Lijn, F., Stokking, R., Ikram, M.A., Vernooij, M.W., Breteler, M.M.B., Niessen, W.J., 2007. Multi-spectral brain tissue segmentation using automatically trained k-Nearest-Neighbor classification. Neuroimage 37, 71–81. https://doi.org/10.1016/j.neuroimage.2007.05.018

Wardlaw, J.M., Bastin, M.E., Valdés Hernández, M.C., Maniega, S.M., Royle, N.A., Morris, Z., Clayden, J.D., Sandeman, E.M., Eadie, E., Murray, C., Starr, J.M., Deary, I.J., 2011. Brain Aging, Cognition in Youth and Old Age and Vascular Disease in the Lothian Birth Cohort 1936: Rationale, Design and Methodology of the Imaging Protocol. International Journal of Stroke 6, 547–559. https://doi.org/10.1111/j.1747-4949.2011.00683.x

Weaver, N.A., Kuijf, H.J., Aben, H.P., Abrigo, J., Bae, H.J., Barbay, M., Best, J.G., Bordet, R., Chappell, F.M., Chen, C.P.L.H., Dondaine, T., van der Giessen, R.S., Godefroy, O., Gyanwali, B., Hamilton, O.K.L., Hilal, S., Huenges Wajer, I.M.C., Kang, Y., Kappelle, L.J., Kim, B.J., Köhler, S., de Kort, P.L.M., Koudstaal, P.J., Kuchcinski, G., Lam, B.Y.K., Lee, B.C., Lee, K.J., Lim, J.S., Lopes, R., Makin, S.D.J., Mendyk, A.M., Mok, V.C.T., Oh, M.S., van Oostenbrugge, R.J., Roussel, M., Shi, L., Staals, J., del C Valdés-Hernández, M., Venketasubramanian, N., Verhey, F.R.J., Wardlaw, J.M., Werring, D.J., Xin, X., Yu, K.H., van Zandvoort, M.J.E., Zhao, L., Biesbroek, J.M., Biessels, G.J., 2021. Strategic infarct locations for post-stroke cognitive impairment:

a pooled analysis of individual patient data from 12 acute ischaemic stroke cohorts. Lancet Neurol 20, 448–459. https://doi.org/10.1016/S1474-4422(21)00060-0

Wen, W., Sachdev, P.S., 2004. Extent and Distribution of White Matter Hyperintensities in Stroke Patients. Stroke 35, 2813–2819. https://doi.org/10.1161/01.STR.0000147034.25760.3d

Wen, W., Sachdev, P.S., Li, J.J., Chen, X., Anstey, K.J., 2009. White matter hyperintensities in the forties: Their prevalence and topography in an epidemiological sample aged 44–48. Hum Brain Mapp 30, 1155–1167. https://doi.org/10.1002/hbm.20586

Wong, A., Law, L.S.N., Liu, W., Wang, Z., Lo, E.S.K., Lau, A., Wong, L.K.S., Mok, V.C.T., 2015. Montreal Cognitive Assessment. Stroke 46, 3547–3550. https://doi.org/10.1161/STROKEAHA.115.011226
